# Pathologic findings and causes of death of stranded cetaceans in the Canary Islands (2006-2012)

**DOI:** 10.1371/journal.pone.0204444

**Published:** 2018-10-05

**Authors:** Josué Díaz-Delgado, Antonio Fernández, Eva Sierra, Simona Sacchini, Marisa Andrada, Ana Isabel Vela, Óscar Quesada-Canales, Yania Paz, Daniele Zucca, Kátia Groch, Manuel Arbelo

**Affiliations:** 1 Veterinary Histology and Pathology, Institute of Animal Health and Food Hygiene (IUSA), University of Las Palmas of Gran Canaria, Las Palmas of Gran Canaria, Spain; 2 Wildlife Comparative Pathology Laboratory, School of Veterinary Medicine and Animal Science, University of São Paulo, São Paulo, Brazil; 3 Department of Animal Health, Veterinary College, Complutense University, Madrid, Spain; 4 Centro de Vigilancia Sanitaria Veterinaria (VISAVET). Complutense University, Madrid, Spain; Animal Health Centre, CANADA

## Abstract

This study describes the pathologic findings and most probable causes of death (CD) of 224 cetaceans stranded along the coastline of the Canary Islands (Spain) over a 7-year period, 2006–2012. Most probable CD, grouped as pathologic categories (PCs), was identified in 208/224 (92.8%) examined animals. Within natural PCs, those associated with good nutritional status represented 70/208 (33.6%), whereas, those associated with significant loss of nutritional status represented 49/208 (23.5%). Fatal intra- and interspecific traumatic interactions were 37/208 (17.8%). Vessel collisions included 24/208 (11.5%). Neonatal/perinatal pathology involved 13/208 (6.2%). Fatal interaction with fishing activities comprised 10/208 (4.8%). Within anthropogenic PCs, foreign body-associated pathology represented 5/208 (2.4%). A CD could not be determined in 16/208 (7.7%) cases. Natural PCs were dominated by infectious and parasitic disease processes. Herein, our results suggest that between 2006 and 2012, in the Canary Islands, direct human activity appeared responsible for 19% of cetaceans deaths, while natural pathologies accounted for 81%. These results, integrating novel findings and published reports, aid in delineating baseline knowledge on cetacean pathology and may be of value to rehabilitators, caregivers, diagnosticians and future conservation policies.

## Introduction

The occurrence of disease in aquatic organisms will be probably one of the long-term consequences of climate change and environmental degradation [1]. Cetaceans are regarded as sentinel species to monitor marine and marine-terrestrial interface ecosystems wherein humans are strictly integrated [1]. Cetaceans are exposed to environmental stressors either anthropogenic, *e*.*g*., chemical and acoustic pollution, fisheries, maritime traffic, tourism industry, and non-anthropogenic, hereafter ‘natural,’ *e*.*g*., biotoxins, pathogens (bacteria, fungi, parasites, viruses) [2]. Several emerging and reemerging pathogens (EREPs) affecting mysticetes and odontocetes have been recognized over the last decades [1,3]. Some of these pathogens have epizootic potential, are zoonotic or display complex pathogeneses in which biotic, *e*.*g*., genetic stock, immunologic dysfunction, and abiotic, *e*.*g*., chemical pollutants, may play a major role [1,4,5].

Major cetacean EREPs include cetacean morbilliviruses, herpesviruses, poxviruses, marine *Brucella* species, *Toxoplasma gondii*, and *Paracoccidioides brasiliensis* (formerly *Lacazia loboi*) [1,3]. Furthermore, neoplasia associated with viral pathogens, such as *Papillomavirus* have been reported [3]. Also, of utmost public health relevance is the isolation of antibiotic resistant bacteria from free-ranging and captive cetaceans [6]. All these pathogens may have the capability to modulate environmental change, mediating decline of species, ecological proportions of predators, prey, competitors, and recyclers; and alter habitat already threatened by habitat fragmentation and climate change [7]. Additionally, novel pathogens have been identified over the last years such as polyomavirus [8], adenovirus [9] and parainfluenza [10], among others. The constantly evolving interplay between marine hosts, pathogens and environmental scenarios demands continuous health and disease monitoring.

The Canary archipelago (Spain) has the greatest cetacean biodiversity of the European coastline with 30 species described, including 7 mysticetes and 23 odontocetes [11]. These include migratory, seasonal and resident species. The first systematic, large-scale and long-term (1999–2005) pathology-based study on free-ranging cetaceans off Canarian waters suggested direct human activity was responsible for 33% of cetaceans deaths, while 62% involved natural disease processes [12]. Our multidisciplinary research team is committed to prolonged and continuous monitoring of health and disease aspects of free-ranging cetaceans off the Canaries; thus, the present study aimed to report the pathologic findings and most probable causes of deaths on cetaceans stranded along the coastline of the Canary Islands between January 2006 to December 2012.

## Materials and methods

The required permission for the management of stranded cetaceans anywhere within the Canarian archipelago was issued by the environmental department of the Canary Islands’ Government. No experiments were performed on live animals because our work was based on dead stranded cetaceans. Biological and stranding epidemiology data for each individual were recorded (S1 Table). Age category was based on total body length and gonadal development, including: fetus/neonate/calf, juvenile/subadult, and adult [13]. The nutritional status (syn. body condition) was subjectively classified into good, moderate, poor, and emaciated according to anatomical parameters such as the osseous prominence of the spinous and transverse vertebral processes and ribs, the mass of the epaxial musculature, and the amount of fat deposits, taking into account the species and the age of the animal [12]. Carcasses were classified as very fresh, fresh, moderate autolysis, advanced autolysis or very advanced autolysis [14].

Necropsies followed standardized protocols [13,14]. Representative samples of skin, *longissimus dorsi* and *rectus abdominis* muscles, peritoneum, diaphragm, central nervous system, eye, pterygoid sac, tympanoperiotic complexes, tongue, oral mucosa, pharyngeal and laryngeal tonsils, esophagus, stomach, small and large intestine, liver, pancreas, trachea, lung, heart, aorta, kidney, ureter, urinary bladder, urethra, lymph nodes, spleen, testicle, penis, prepuce, ovary, uterus, vagina and vulva, were collected and fixed in 10% neutral buffered formalin. All these tissues were processed routinely, embedded in paraffin-wax and 5 μm-thick sections were stained with hematoxylin and eosin (H&E) for microscopic analysis. Special histochemical techniques (4–10 μm-thick sections) to better characterize microscopic changes on selected tissue sections included: periodic acid-Schiff, luxol fast blue, Prussian blue, Bielchowsky’s, Gram/Twort, Hall’s, Grocott- Gomori’s Methenamine Silver, Masson’s trichrome, Movat-Russel pentachrome, osmium tetroxide (post-fixation), rhodamine, Congo red, von Kossa, and Ziehl-Neelsen.

We employed a set of primary antibodies for immunohistochemical (IHC) analysis to better define the nature of some lesions in certain animals. Primary antibodies utilized were: calponin, calretinin, cluster of differentiation (CD)-3, CD79α, cytokeratin (CK)-7, CK14, CK20, glial fibrillary acid protein (GFAP), lysozyme, myeloid/histiocyte antigen (MAC)-387, neuron specific enolase (NSE), neurofilament, pancytokeratin AE1/AE3, S100 protein, and vimentin. When indicated by histopathology or observation of specific etiologic agents in sections, targeted infectious agents, *i*.*e*., cetacean morbillivirus (CeMV), Herpesvirus (HV), *T*. *gondii*, and *Erysipelothrix rhusiopathiae*, via IHC was undertaken. Published methodologies for IHC analyses were followed [15,16]. Detailed information on IHC methodologies are recorded in S2 Table. Negative controls consisted of serial tissue sections in which primary antibodies were substituted by non-immune homologous serum. Positive controls included appropriate cetacean, canine and/or human tissue sections, accordingly.

Fresh tissue samples (skin, muscle, lung, prescapular, pulmonary, mediastinal and mesenteric lymph nodes, liver, intestine, kidney, spleen, brain) collected routinely during necropsy, were frozen (-80°C) and selectively submitted for bacteriological analysis. These included routine culture and surface plating on routine media, *e*.*g*., Columbia blood agar and preliminary identification of isolates via API system (API 20E, API Rapid 20E, API Staph, API 20 Strep, API Coryne, API 20A). PCR targeting the 16S rRNA gene coupled with pulsed-field gel electrophoresis were performed on selected isolates [17]. Cerebrum of animal no. 63 was subjected to mycological analysis, including culture on Sabouraud agar and morphologic colony identification [18]. Tissues cultured and respective results are recorded in S3 Table. PCR analysis for detection of CeMV and HV followed published protocols and have been partially published [19,20].

For parasitological analysis, epibionts, ectoparasites and endoparasites were preserved in 70% alcohol. Identification relied on macroscopic, submacroscopic and histologic features [21]. Molecular analysis using primers 930F and 1200R for the 18S ribosomal gene were used in two *Crassicauda* sp. nematodes from animal no. 180 and 207 [22].

The classification herein implemented focuses on differentiating between natural and anthropogenic disease processes, hereafter ‘pathologic categories’ (PCs), according to current knowledge on cetacean pathology [12]. We considered five major natural PCs: 1) pathology associated with significant loss of nutritional status (NPSLNS; including poor and emaciated animals), 2) pathology associated with good nutritional status (NPGNS; including good and moderate animals), 3) neonatal/perinatal pathology (NPP), 4) intra- and interspecific traumatic interactions (ITI), and 5) typical mass-stranding pathology. Three major anthropogenic PCs were considered: 1) interaction with fishing activities (IFA), 2) foreign body-associated pathology (FBAP), and 3) vessel collision (VC) [12]. Furthermore, gross and microscopic lesions typically associated with the ‘stress response syndrome’ or ‘alarm reaction’ [23] and ‘capture myopathy’ [24,25] were also addressed in this classification system [12].

## Material access

The material used in this study is deposited in the Marine Mammal Tissue Bank held by the Animal Histology and Pathology division of the Institute for Animal Health and Food Hygiene, Veterinary School, University of Las Palmas of Gran Canaria, Gran Canaria, Spain.

## Results

### Stranding epidemiology

A total of 320 cetaceans stranded between 1^st^ January, 2006, and 31^st^ December, 2012, including 22 species. From these, postmortem examinations were conducted on 224 (70%), representing 21 species. Odontocetes (n = 216; 17 species) were overrepresented compared to mysticetes (n = 8; four species) (S1 Table). Sex distribution of animals studied included 105 (46.9%) females, 109 (48.7%) males, and 10 (4.5%) undetermined. Age categories were: 51/224 (22.8%) neonate/calf, 59/224 (26.3%) juvenile/subadult and 113/224 (50.4%) adult. Age was unknown in one animal. Annual strandings during this 7-year period averaged 45.7; strandings occurred throughout the year with peaks between March, April and May. Necropsies were performed on 13/224 (5.8%) very fresh, 79/224 (35.3%) fresh, 61/224 (27.2%) moderate autolysis, 60/224 (26.8%) advanced autolysis and 11/224 (4.9%) very advanced autolysis carcasses. Thirty of 224 (13.4%) animals stranded alive; 194/224 (86.6%) stranded dead or were retrieved adrift. Distribution based on nutritional status included: 56 (25%) good, 60 (26.8%) moderate, 64 (28.6%) poor and 16 (7.1%) cachectic animals. The nutritional status could not be inferred in 28 (12.5%) animals due to decomposition phenomena.

The most probable CD, grouped in PC s, was identified in 208/224 (92.8%) examined individuals (Table 1). Etiologic diagnoses by pathologic categories are recorded in Table 2.

**Table 1:** Species studied and pathologic categories in stranded cetaceans from the Canary Islands (2006–2012).

	Pathologic categories							ND	Total
Species	Natural				Anthropogenic				
	PSLNS	PGNS	NPP	IITI	FBAP	VC	IFA		
*Balaenoptera acutorostrata*	0	0	1	1	0	1	0	0	3
*Balaenoptera borealis*	1	1	0	0	0	0	0	1	3
*Balaenoptera physalus*	0	0	0	0	0	1	0	0	1
*Delphinus delphis*	9	9	0	3	0	0	1	2	24
*Globicephala macrorhynchus*	3	8	3	8*	0	1	1	2	26
*Grampus griseus*	5	1	0	3	1	0	0	0	10
*Kogia breviceps*	3	3	1	4	0	4	0	1	16
*Kogia sima*	0	1	0	0	0	0	0	1	2
*Lagenodelphis hosei*	0	0	0	0	0	0	1	0	1
*Megaptera novaeangliae*	0	0	0	0	0	1	0	0	1
*Mesoplodon bidens*	0	0	0	0	1	0	0	0	1
*Mesoplodon europaeus*	0	0	0	1	1	3	0	2	7
*Mesoplodon mirus*	0	0	0	1	0	0	0	0	1
*Phocoena phocoena*	0	0	1	0	0	0	0	0	1
*Physeter macrocephalus*	0	1	1	2	0	11*	0	1	16
*Pseudorca crassidens*	0	1	0	1	0	0	0	0	2
*Stenella coeruleoalba*	18*	14	4*	4	0	0	2	1	43
*Stenella frontalis*	6	16*	2	4	1	0	4*	3	36
*Steno bredanensis*	2	1	0	0	0	0	1	0	4
*Tursiops truncatus*	2	9	0	4	0	0	0	2	17
*Ziphius cavirostris*	0	5	0	1	1	2	0	0	9
**Total**	49	70	13	37	5	24	10	16	224

PSLNS: pathology associated with significant loss of nutrional status; PGNS: pathology with good nutritional status; NPP: neonatal-perinatal pathology; IITI: intra-/interspecific traumatic interactions; FBAP: foreign body-associated pathology; VC: vessel collisions; IFA: interaction with fishing activities. ND: not determined.

*Species most affected in each category.

**Table 2:** Pathologic categories and etiologic diagnoses for cetaceans studied that stranded in the Canary Islands (2006–2012).

Pathologic category	Etiologic diagnosis	No
**Natural**		
Pathology associated with good nutritional status	Infectious (non-parasitic) disease	42
	Parasitic disease	20
	Gas embolism	2
	Encephalopathy (unknown origin)	1
	Neoplasia	1
	Idiopathic hemorrhage	1
	Uterine rupture	1
	Senile changes	1
	Intestinal torsion	1
Pathology associated with significant loss of nutritional status	Infectious (non-parasitic) disease	33
	Parasitic disease	13
	Neoplasia	1
	Senile changes	1
	Trauma	1
Intra- and interespecific traumatic interactions	Traumata, bite scars and/or tooth-rake marks	36
	Gas embolism	1
Neonatal or perinatal pathology	Fetal distress	6
	Dystocia	2
	Infectious	2
	Neonatal weakness	1
	Abortion (prematurity)	1
	Developmental anomalies	1
**Anthropogenic**		
Vessel collision	Keel or propeller sharp trauma with/without blunt traumata	24
Interaction with fishing activities	Blunt trauma	6
	Bycatch	2
	Fishing tool sharp trauma	2
Foreign body-associated pathology	Foreign body ingestion	4
	Foreign body entanglement (presumed floating net)	1

### 1. Natural pathologic categories

#### 1.1 Pathology associated with good nutritional status

NPGNS involved 70/208 (33.6%) animals, representing 13 species (Table 1). The main morphologic diagnoses for NPGNS cases are recorded in S4 Table. Etiologic diagnoses identified were: infectious (60%), parasitic (28.6%), other (gas encephalopathy, idiopathic hemorrhage, uterine rupture; 5.7%), gas embolism (2.8%), neoplasia (1.4%), and senile changes (1.4%).

**Fig 1:**
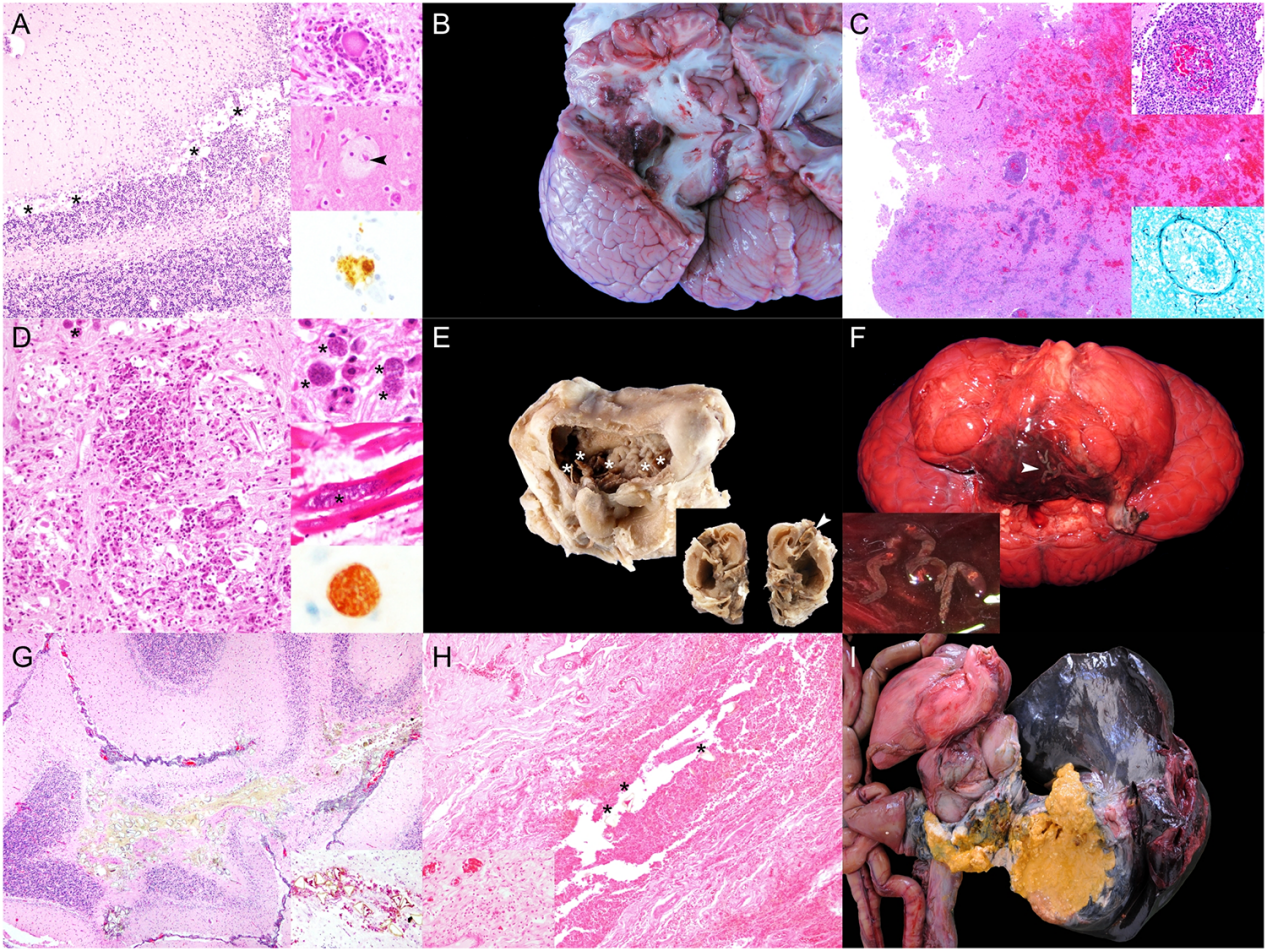
Panel of ‘natural pathologies associated with good nutritional status’ in cetaceans stranded in the Canary Islands (2006–2012). A) Cetacean morbillivirus infection (animal no. 202; *Stenella coeruleoalba*). There is severe mononuclear inflammation at the cerebellar foliar grey and white matter interface with rarefaction at the Purkinje cell layer (asterisks). Hematoxylin and eosin (H&E). Right upper inset: focal neuronal degeneration and neuronophagia. H&E. Right middle inset: Swollen and chromatolytic cortical neuron with intranuclear eosinophilic inclusion body (arrowhead). Right lower inset: Degenerating neuron shows strong intranuclear and intracytoplasmic granular morbilliviral immunolabeling. Immunohistochemistry (IHC) for CDV. B) *Aspergillus fumigatus* encephalitis (animal no. 63; *S*. *coeruleoalba*). Multifocal to coalescing necrohemorrhagic encephalitis in the left cerebral hemisphere (asterisk). C) *Aspergillus fumigatus* encephalitis (animal no. 63; *S*. *coeruleoalba*). Severe pleocellular and necrohemorrhagic cerebral inflammation. H&E. Right upper inset: focal necrotizing vasculitis with hemorrhage and perivascular cuffing. H&E. Right lower inset: Necrotizing vasculitis with intralesional hyphae. Gomori Methenamine-Silver nitrate (GMS). D) Systemic toxoplasmosis (animal no. 46; *S*. *frontalis*). Severe lymphohistiocytic to granulomatous white matter inflammation with neuroparenchyma necrosis and rarefaction and rare *T*. *gondii* protozoal cysts (asterisk). H&E. Right upper inset: some protozoal cysts had no evident inflammation (asterisks). Right middle inset: *T*. *gondii* cysts within sarcoplasm of cardiomyocytes (asterisk). H&E. Right lower inset: cerebral protozoal cyst is strongly positive for anti-*T*. *gondii* antibody. IHC for *T*. *gondii*. E) Middle and inner ear nasitremiasis (animal no. 220; *Tursiops truncatus*). Lateral window (removed) of tympanic bulla (middle ear) after fixation and decalcification. There is marked proliferative otitis media with numerous intralesional adult *Nasitrema* sp. (asterisks). Inset: longitudinal section through the cochlea (inner ear). The cochlear nerve is swollen, dark and fragmented (arrowhead). F) Rhombencephalic nasitremiasis (animal no. 220; *T*. *truncatus*). Ventral surface of brainstem shows locally extensive necrotizing and hemorrhagic meningoencephalitis with intralesional adult *Nasitrema* sp. (arrowhead). Inset: detail of adult *Nasitrema* sp. G) Cerebellar nasitremiasis (animal no. 157; *T*. *truncatus*). Pyogranulomatous and necrotizing encephalitis with intralesional *Nasitrema* sp. eggs. H&E. Inset: Detail of pleocellular pyogranulomatous inflammation and *Nasitrema* sp. eggs. Periodic acid–Schiff (PAS). H) Inner ear nasitremiasis (animal no. 220; *T*. *truncatus*). Pyogranulomatous and necrotizing cochlear neuritis with intralesional *Nasitrema* sp. eggs (asterisks). H&E. Inset: endoneural lymphoplasmacytic inflammatory infiltrate in proximal cochlear nerve. H&E. I) Gastrohepatic abscess (animal no. 208; *S*. *coeruleoalba*). Severe transmural pyogranulomatous gastritis and hepatitis leading to large right hepatic lobe abscess.

CeMV infection (Fig 1a) was confirmed via IHC and/or PCR analysis in six animals: two short-finned pilot whales (*Globicephala macrorhynchus*; no. 17 and 172), two striped dolphins (*Stenella coeruleoalba*; no. 152 and 202), one Atlantic spotted dolphin (*Stenella frontalis*; no. 126), and one short-beaked common dolphin (*Delphinus delphis*; no. 36). These animals often had multicentric lymphoid depletion. Furthermore, 14% (10/70) of NPGNS animals presented varying degrees of central nervous system (CNS) inflammation; however, the etiology was not apparent ‘unknown origin meningoencephalitides’.

Septicemia by *E*. *rhusiopathiae* was diagnosed in an Atlantic bottlenose dolphin and an Atlantic spotted dolphin (animal no.133 and 189) [17]. Septicemia by *Streptococcus phocae* was diagnosed in an adult female short-beaked common dolphin (animal no. 36) [26]. Fungal encephalitis by *Aspergillus fumigatus* (Fig 1b and 1c) was observed in a male calf striped dolphin (animal no. 63) stranded alive. PCR analyses for CeMV and HV in cerebrum were negative.

Four Atlantic spotted dolphins including three adults and one calf (animal no. 7, 46, 126 and 131) had toxoplasmosis. Most severe and extensive lesions in these cases were confined to the CNS (Fig 1d); however, there was also involvement of the heart, adrenal glands and gastrointestinal system in animal no. 46, 126 and 131 Severe arterial and renal crassicaudiasis was diagnosed in five Cuvier’s beaked whales (CBW; *Ziphius cavirostris*) (animal no. 92, 118, 180, 167 and 207) [22]. Severe pterygoid sinusitis by *Nasitrema* sp. with middle and inner ear involvement (Fig 1e) and extension to the CNS (Fig 1f and 1g) was observed in three Atlantic bottlenose dolphins (*Tursiops truncatus*) (animal no. 157, 220 and 224) and one short-beaked common dolphin (animal no. 195). Inflammatory changes included pyogranulomatous and necrotizing meningoencephalomyelitis, meningomyelitis and vestibulocochlear neuritis (Fig 1h) with intralesional adult and trematode ova, spongiosis, vascular necrosis and vasculitis. Animal no. 224 also presented intralesional Gram-positive bacilli; bacterial culture of cerebrum yielded *Clostridium* sp.

An adult, female striped dolphin (animal no. 208) presented a large hepatic abscess [analogous to case 134, NPSLNS], firmly adhered to the pyloric stomach wall (Fig 1i). The abscess ruptured and led to septic peritonitis. Severe parasitization by *Brachycladium atlanticum* and *Pholeter gastrophilus* in the biliary tract and the pyloric stomach, respectively, were associated with the ruptured abscess and adjacent inflamed areas. Severe *Bolbosoma* sp. intestinal parasitization was noted in an adult female sei whale (*Balaenoptera borealis*) (animal no. 188). Numerous acanthocephalans were embedded in the submucosa and led to complete luminal obliteration and hemorrhage. Peritoneal migrating *Bolbosoma* sp. were common. Similarly, severe *Bolbosoma* sp. parasitization was observed in a false killer whale (*Pseudorca crassidens*) (animal no. 192); however, hemorrhage was not a major component. Severe *Crassicauda* sp. parasitization affecting the cervical and thoracic subcutis, fascia, and muscle, the cervical gland and the rete mirabile was determined in an adult female pigmy sperm whale (*Kogia breviceps*) (animal no. 198). Additionally, *Crassicauda* sp. was associated with massive urethral infestation in an Atlantic spotted dolphin (animal no. 83).

Fatal gas embolism was diagnosed in two animals (animal no.147 and 163) [27,28]. A thalamic high-grade astrocytoma (glioblastoma multiforme) was diagnosed in an adult male Atlantic spotted dolphin (animal no. 50) [29]. A live-stranded adult male pigmy sperm whale (animal no. 56), evidenced severe spongiosis of the neuroparenchyma, mainly in the brainstem, thalamus and cerebral white matter. Other microscopic changes in these areas were axonal spheroids, cytotoxic and perivascular edema, astrocytosis, Alzheimer type II-like astrocytes, Gitter cells and scattered neuronal satellitosis in the cerebral cortex and thalamus. These changes were bilateral and asymmetrical. No demyelination or axonal anomalies were observed by means of luxol fast blue and Bielchowsky’s stains, respectively. Other unusual etiologic diagnoses associated with single deaths included uterine rupture (animal no. 191) and segmental intestinal torsion with mesenteric venous infarction, intestinal ischemic necrosis and luminal hemorrhage (animal no. 65).

#### 1.2 Pathology associated with significant loss of nutritional status

NPSLNS involved 49/208 (23.5%) animals, representing 9 species (Table 1). The main morphologic diagnoses for NPSLNS cases are recorded in S5 Table. Etiologic diagnoses identified were: infectious (69.4%), parasitic (26.5%), neoplasia (2.0%), and senile changes (2.0%).

**Fig 2:**
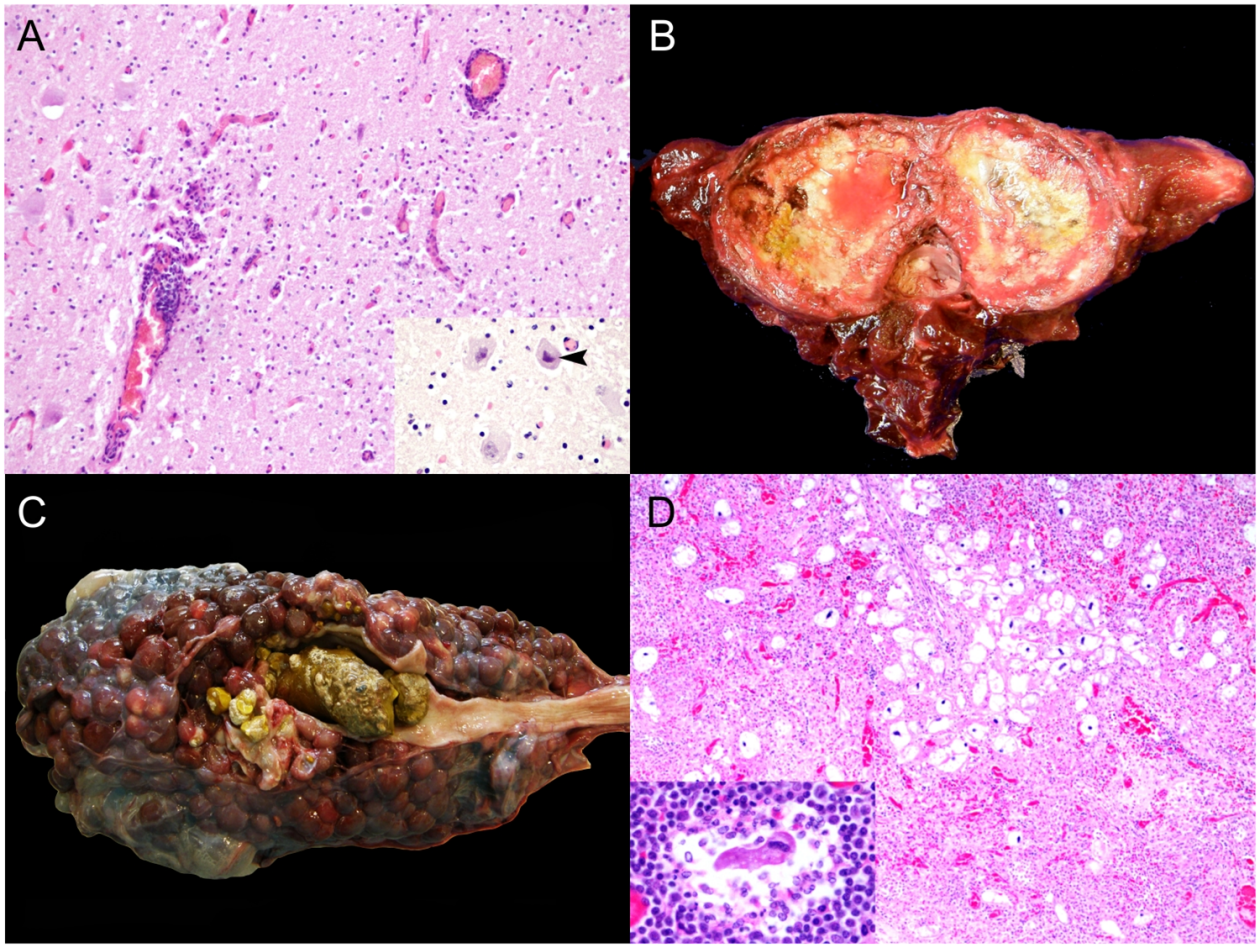
Panel of natural pathologies associated with loss of nutritional status in cetaceans stranded in the Canary Islands (2006–2012). A) Cerebral herpesvirus infection (animal no. 165; *S*. *coeruleoalba*). Lymphocytic encephalitis with perivascular cuffing and gliosis. H&E. Inset: Focal eosinophilic intranuclear inclusion body (arrowhead). H&E. B) Atlanto-occipital osteoarthritis (animal no. 165; *S*. *coeruleoalba*). Marked, chronic osteoarthritis and synovitis with cartilage loss at the cranial articular facets of atlas vertebra. C) Nephrolithiasis (animal no. 127; *Delphinus delphis*). Severe nephrolithiasis with hydronephrosis, proximal hydroureter and chronic renicular disease with atrophy and hypertrophy. D) Ciliate protozoal lymphadenitis (animal no. 94; *S*. *bredanensis*). Pyogranulomatous and necrotizing prescapular lymphadenitis with numerous intralesional ciliate protozoan. H&E. Inset: Detail of ciliate protozoon surrounded by neutrophils and necrotic cell debris, and lymphocytes. H&E.

Among infectious disease processes, the CNS was commonly targeted. Viral agents identified were CeMV and HV, as determined by IHC and/or PCR analysis. Five odontocetes of three species: three striped dolphins (animal no. 43, 61 and 97), one Risso’s dolphins (*Grampus griseus*) (animal no. 77), and one short-finned pilot whale (animal no. 183) presented positive immunolabeling and/or a positive PCR result for CeMV. Cerebral HV infection (Fig 2a) was diagnosed in an adult male striped dolphin (animal no. 165) and confirmed by PCR [19]. Additionally, *Mycoplasma* sp. was isolated from the atlanto-occipital joint, which displayed severe, chronic osteoarthritis (Fig 2b). About 30.6% (15/49) of the NPSLNS were meningoencephalitides of undetermined etiology.

A juvenile female rough-toothed dolphin (*Steno bredanensis*) (animal no. 68) presented ulcerative glossitis, palatitis, esophagitis and gastritis, and dermatitis with scattered eosinophilic intranuclear inclusion bodies, highly suggestive of HV infection. Numerous intravascular coccobacilli, leukocytosis and disseminated intravascular coagulation were also noted. Bacteriologic cultures yielded *Pseudomonas taetrolens* (liver, brain); *Aeromonas hydrophila*/*caviae* (kidney, lung); *P*. *putida* (lung, brain); and *A*. *salmonicida* (brain). *Wohlfahrtiimonas chitiniclastica* septicemia was diagnosed in an adult male short-beaked common dolphin (animal no. 213) [30]. *Pseudomonas aeruginosa* was isolated from renal tissue in an adult male short-beaked common dolphin (animal no. 127) with severe nephrolithiasis (Fig 2c) and ureterolithiasis. Microscopically, there was suppurative pyelonephritis with tubular necrosis and Gram-negative bacilli, ischemic necrosis (infarcts), interstitial fibrosis and tubular proteinosis. Additionally, mild multifocal lymphocytic meningitis with occasional intravascular Gram-negative bacilli and lymphoplasmacytic cortical adrenalitis were seen.

Other interesting cases with severe and extensive lesions suggestive of systemic infectious disease albeit without confirmed etiologies involved an adult pigmy sperm whale (animal no. 6), an adult female striped dolphin (animal no. 54), and an adult male striped dolphin (animal no. 110). All these animals presented septic peritonitis. The first presented a large hepatic abscess that ruptured and led to peritonitis and hemoabdomen. An incidental hepatocellular carcinoma was also observed. The second animal had septic peritonitis due to a perforating ulcer in the keratinized stomach. The third animal presented peritonitis along with pyothorax/empyema, fibrinosuppurative pleuropneumonia and pericarditis.

Disseminated toxoplasmosis was observed in a female calf Atlantic spotted dolphin (animal no. 96). An adult male short-beaked common dolphin (animal no. 45) and a subadult male common bottlenose dolphin (animal no. 184), presented with severe and extensive pterygoid sinusitis, otitis and meningoencephalitis due to *Nasitrema* sp. trematodes. *Nasitrema delphini* [31] was identified in animal no. 184. Animal no. 94 had multisystemic ciliate protozoosis characterized by multifocal pyogranulomatous and ulcerative dermatitis and panniculitis with marked necrosis, hemorrhage and numerous ciliated protozoa; multicentric pyogranulomatous lymphadenitis with ciliated protozoa (Fig 2d); and histiocytic and neutrophilic encephalitis with necrosis and hemorrhage.

A primary uterine T cell lymphoma with disseminated metastasis was observed in an adult female Atlantic spotted dolphin (animal no. 49) [32].

#### 1.3 Neonatal/Perinatal pathology

NPP was determined in 13/208 (6.2%), representing 7 species (Table 1). The main morphologic diagnoses for animals included in this category are shown in S6 Table. Etiologic diagnoses identified were: fetal distress (46%), dystocia (15%), infectious (15%), abortion (7.7%), congenital malformation (7.7%), and neonatal maternal-filial separation/maternal neglect; 7.7%.

**Fig 3:**
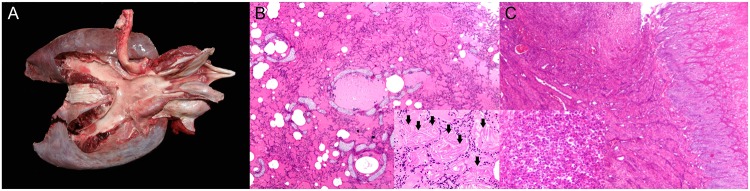
Panel of neonatal/perinatal pathologies in cetaceans stranded in the Canary Islands (2006–2012). A) Pulmonary edema (animal no. 132; *Balaenoptera acutorostrata*). The trachea, mainstem bronchi and primary bronchi are filled with abundant frothy fluid. B) Pulmonary edema (animal no. 21; *Stenella frontalis*). The bronchioles and alveoli are diffusely filled by abundant proteinaceous fluid. Inset: numerous keratin squames (arrows) fill in the alveoli. H&E. C) Omphaloarteritis (animal no. 213; *Kogia breviceps*). The umbilical margin is infiltrated by abundant pleocellular suppurative inflammatory cells and fibrin. H&E. Inset: Numerous viable and degenerate neutrophils with macrophages, edema, fibrin and necrotic cell debris. H&E.

All animals with presumed fetal distress (animal no. 21, 23, 29, 38, 121 and 176) had moderate to severe pulmonary edema (Fig 3a and 3b) with individualized intraalveolar, intrabronchiolar and intrabronchial squames or most often large aggregates of aspirated keratinized stratified epithelium with retained nuclei. Other common findings were diffuse pulmonary atelectasis with alternate areas of emphysema, bronchodilation, bronchiolar constriction, and intraalveolar foamy macrophages. Animal no. 23 had bronchointerstitial pneumonia with intraalveolar and intrabronchiolar meconium along with squames and alveolar histiocytosis. Animal no. 121 also presented alveolar proteinosis and hyaline membranes. Animal no. 88, with presumed dystocia, had multifocal extensive areas of congestion and hemorrhage, lineal to circumferential, typically in the subcutis of the mandibular and cranial regions, bilaterally in the thoracolumbar area, the pectoral flippers and the abdominal wall.

Several animals in this group had evidence of neonatal/perinatal infection. Animal no. 48, a presumably premature short-finned pilot whale (total body length: 130 cm-long; reference range: 140–185 cm) [33] presented findings suggestive of sepsis (multiorgan intravascular bacteria including kidney, lung, pulmonary and prescapular lymph node, spleen and brain, and hemorrhage) and fetal distress. A calf pygmy sperm whale (animal no. 213) presented with fibrinosuppurative omphaloarteritis and phlebitis (Fig 3c), neutrophilic omentitis, and pulmonary edema and hemorrhage. The etiology in these cases was unknown, but bacterial infection was suspected. No cultures were attempted. Additionally, animal no. 30, a striped dolphin calf had several congenital malformations including segmental intestinal atresia and spinal meningocele; however, autolysis limited detailed microscopic examination. There was also lack of development of multiple cranial bones and incomplete closure of fontanels.

#### 1.4 Intra- and interspecific traumatic interactions

IITI involved 37/208 (17.8%) animals, representing 13 species (Table 1). The main morphologic and etiologic diagnoses for IITI cases are recorded in S7 Table.

**Fig 4:**
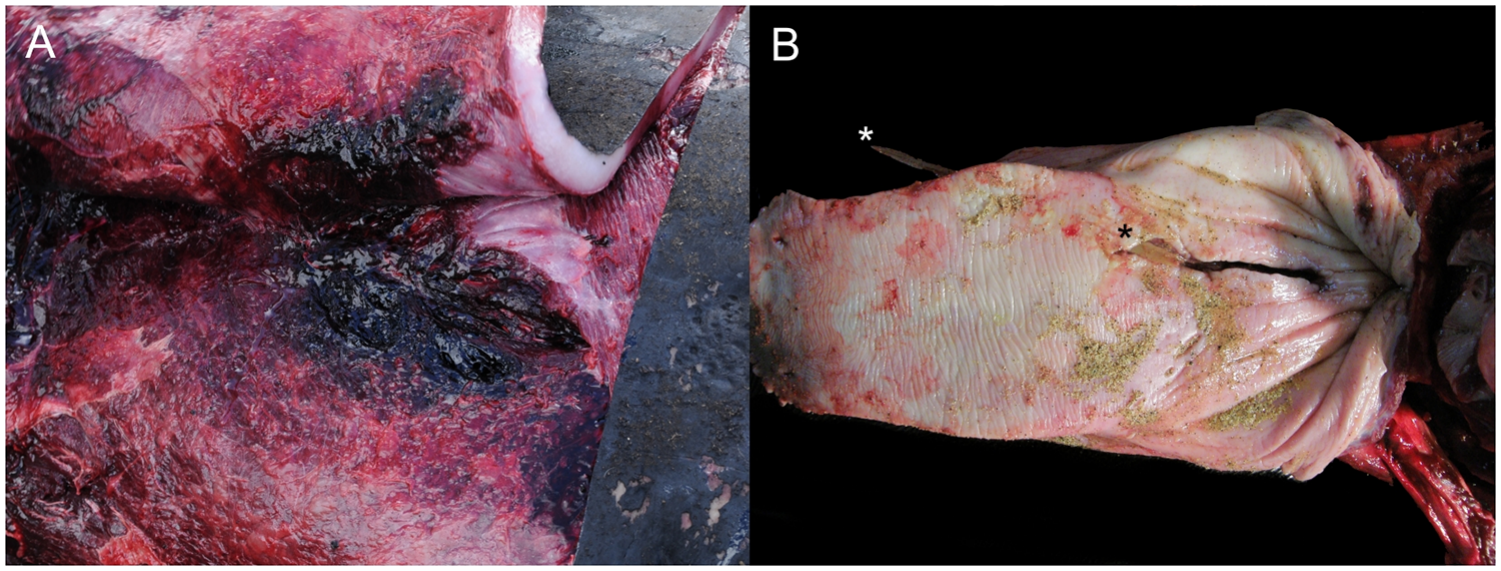
Panel of pathologies associated with fatal intra- and interspecific interactions in cetaceans stranded in the Canary Islands (2006–2012). A) Subcutaneous hemorrhage (animal no. 221; *Mesoplodon mirus*). The subcutis and suprascapular musculature is focally expanded by hemorrhage. B) Traumatic glossitis (animal no. 64; *Pseudorca crassidens*). Full-thickness perforating traumatic glossitis with intralesional stingray spine (asterisks).

Consistent trauma-associated findings in these animals were focally extensive to suffusive hemorrhage, hematomas and myonecrosis (Fig 4a), mostly in the subcutis and axial musculature, but also in internal viscera. Tooth rakes-associated erosions and lacerations were often associated with above vascular changes. Other common findings were fractures of the axial skeleton and intracavitary and visceral hemorrhage. Pulmonary fat emboli and myo-/hemoglobinuric nephrosis were occasionally observed. In some of these cases, underlying inflammatory infectious disease was observed, for instance, meningoencephalomyelitis and proliferative and sclerosing bronchitis (animal no. 123 and 206), disseminated toxoplasmosis (animal no.135), and severe arterial and renal crassicaudiasis (animal no. 211). Furthermore, two unusual interspecific traumatic events were seen. In the first case, a juvenile male false killer whale (animal no. 64) in poor body condition presented severe, focal, chronic perforating glossitis and stomatitis with an intralesional stingray spine (Fig 4b). The second case involved a male adult Risso’s dolphin with systemic gas embolism associated with complicated predatory attempts on squids [28].

### 2. Anthropogenic pathologic categories

#### 2.1 Interaction with fishing activities

Fatal IFA involved 10/208 (4.8%) animals, representing 6 species (Table 1). The main morphologic diagnoses in IFA cases are recorded in S8 Table. Etiologic diagnoses identified were: blunt trauma (60%), fishing tool (*e*.*g*., harpoon, hook, palangre) sharp trauma (20%) and bycatch (20%).

**Fig 5:**
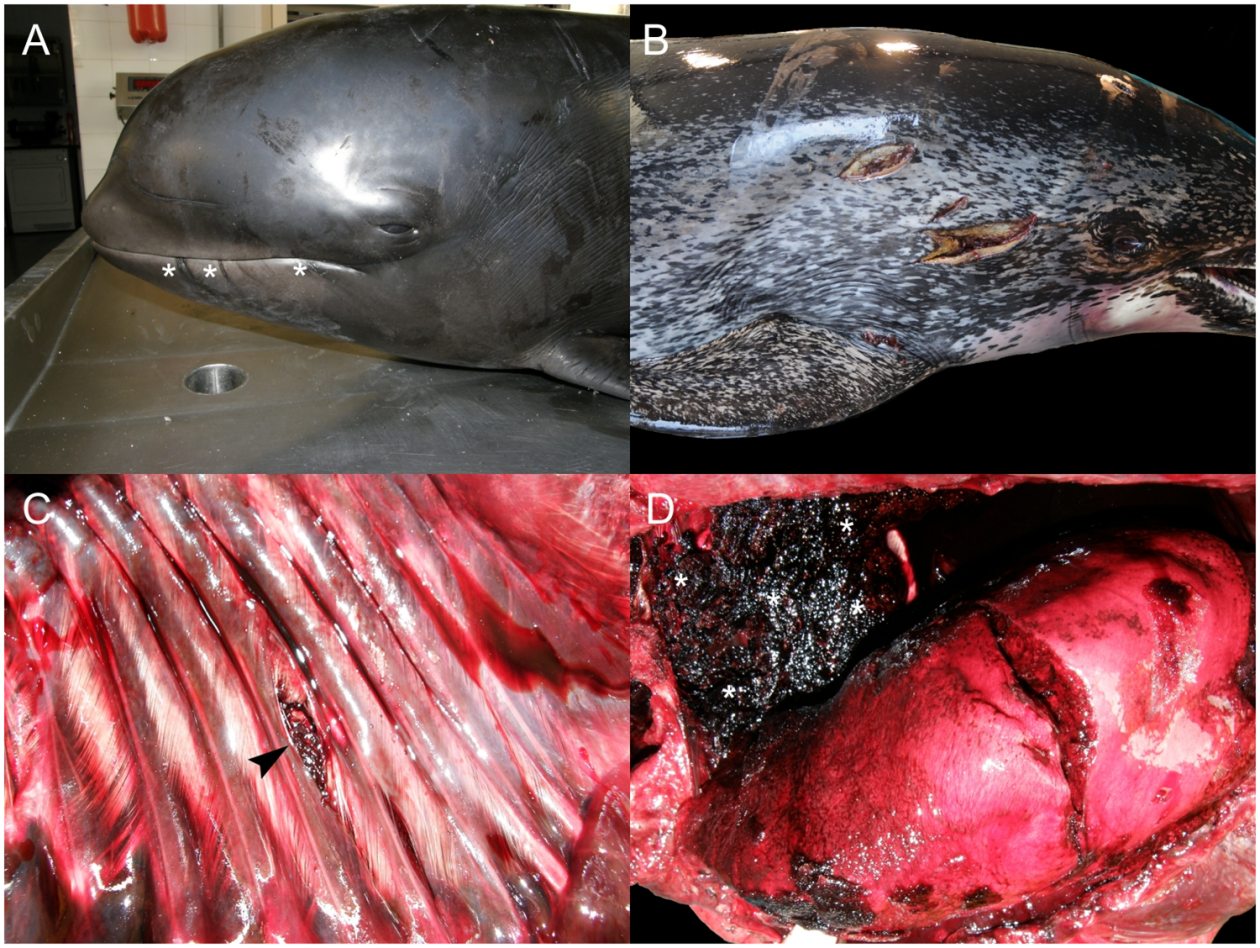
Panel of pathologies associated with fatal interaction with fishing activities in cetaceans stranded in the Canary Islands (2006–2012). A) Fishing net markings (animal no. 57; *Globicephala macrorhynchus*). The left mouth commissure and adjacent mandibular skin has multiple, linear, parallel erosions and lacerations due to net entanglement (asterisks). B) Anthropogenic fishing-related incisive wounds (animal no. 199; *S*. *frontalis*). There are two deep incised skin cuts on the right suprascapular region. C) Thoracic wall perforation (animal no. 66; *S*. *frontalis*). There is a focal mid-diaphyseal rib fracture with muscle tearing and hemorrhage (arrowhead) on the left thoracic wall. D) Lung perforation (animal no. 66; *S*. *frontalis*). The left lung is focally perforated and lacerated and there is abundant hemothorax (asterisks) (there is some freezing artifact).

Bycatch-associated lesions included linear and perpendicular superficial cutaneous erosions and lacerations around the rostrum and gingiva in a neonate female short-fined pilot whale (animal no. 57; Fig 5a), and lacerations in the rostrum, forehead and ventrocaudal aspect of the body along with ulcers on the buccal commissures in an adult female striped dolphin (animal no. 194). Blunt trauma with subcutaneous and cranioencephalic contusions, fractures and hemorrhage were noted in several individuals (animal no. 34, 37 and 71). Furthermore, lesions presumably linked to release from fishing nets were observed in animal no. 34 (Fig 5b). Multiple cutaneous penetrating wounds with right lung perforation and hemothorax were observed in animal no. 66 (Fig 5c and 5d). An Atlantic spotted dolphin (animal no.199) had necrotic stomatitis and mandibular osteomyelitis by perforating hook in addition to multiple cutaneous incisions of presumptive anthropogenic origin.

#### 2.2 Foreign body-associated pathology

FBAP was determined in 5/208 (2.4%) animals, representing 5 species (Table 1). From these, four presented profound emaciation while one of the animals was in good nutritional status. The main morphologic diagnoses in animals included in this category are recorded in S9 Table. Foreign bodies were associated with gastric obstruction and impaction (60%), gastric perforation (20%), and chronic entanglement (20%).

**Fig 6:**
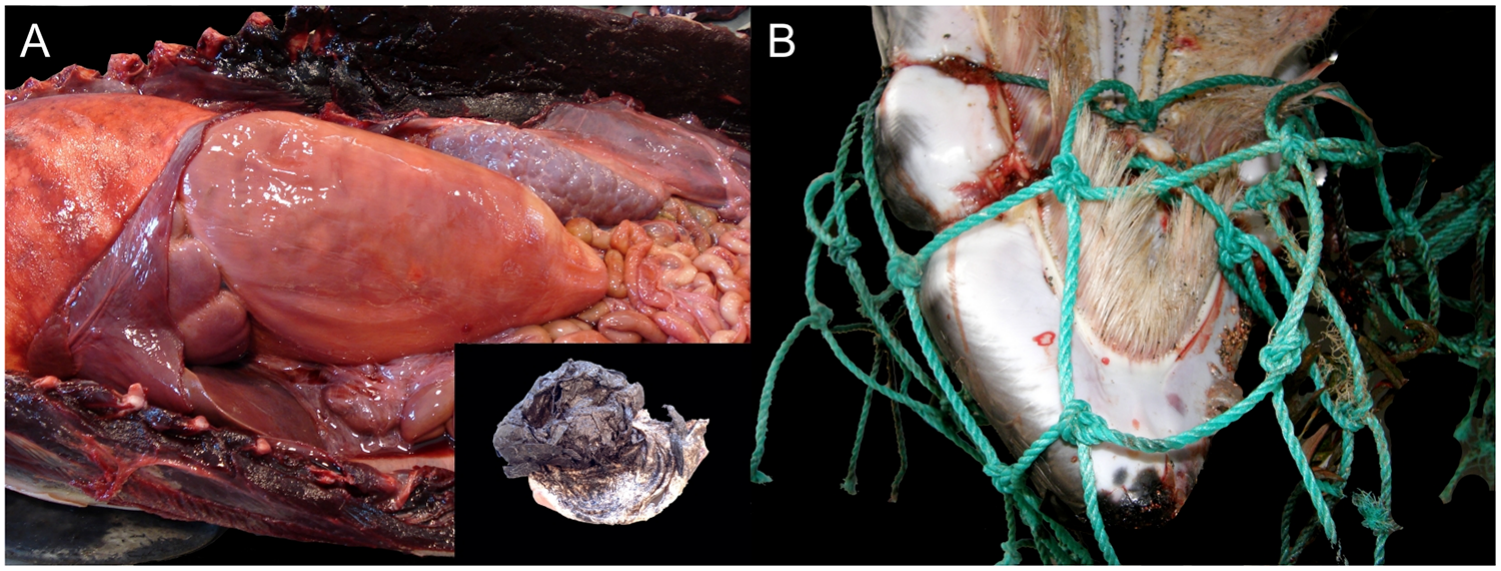
Panel of foreign body-associated pathologies in cetaceans stranded in the Canary Islands (2006–2012). A) Gastric impaction (animal no. 27; *Stenella frontalis*). The keratinized stomach is filled with abundant plastics. Inset: detail of plastic mass mixed melena. B) Chronic entanglement (animal no. 42; *Balaenoptera acutorostrata*). The fishing net had lodged deeply around the maxilla leading to soft tissue constrictive necrosis, periostitis and osteomyelitis (not visible in the image) and exuberant granulation tissue.

In a juvenile female Gervais’ beaked whale (*Mesoplodon europaeus*) (animal no. 13), gastric perforation by a linear foreign body, *i*.*e*., coiled wire, led to septic fibrinosuppurative peritonitis and death. In a juvenile Cuvier’s beaked whale (animal no. 19) and an adult Risso’s dolphin (animal no. 219), a large number of plastics including packing tape, trash bags, and a rope, respectively, had lodged and severely impacted the keratinized gastric compartment. Luminal gastrointestinal hemorrhage was noted in the first animal, whereas the second had multifocal non-bleeding gastric ulcers. An adult female Atlantic spotted dolphin (animal no. 27) presented ulcerative and hemorrhagic gastritis associated with gastric impaction by 20 plastic bags mixed with abundant melena (Fig 6a). Animal no. 107, a common minke whale calf had profound cachexia associated with chronic entanglement linked to fishing net (Fig 6b).

#### 2.3 Vessel collisions

**Fig 7:**
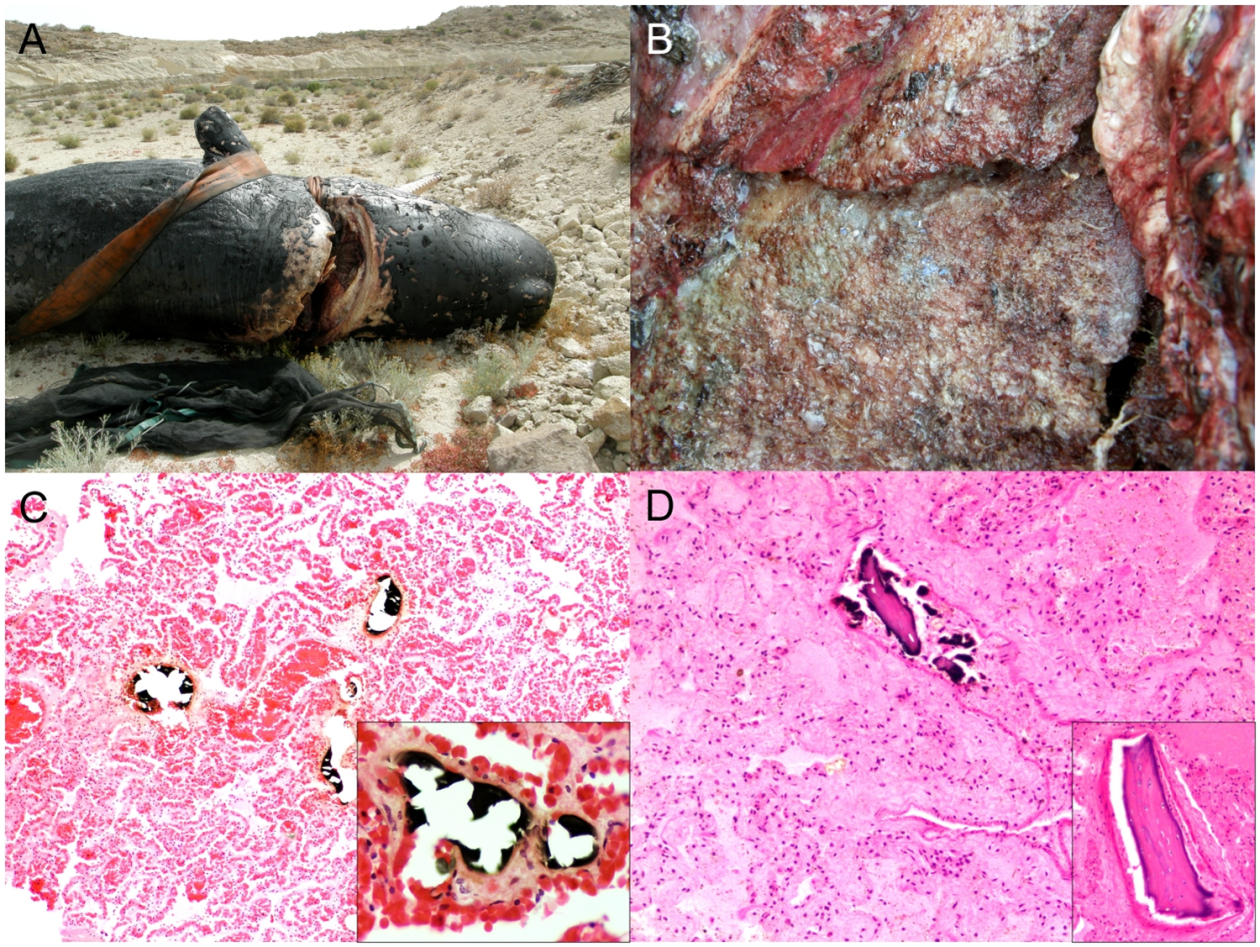
Panel of vessel collision-associated pathologic findings in cetaceans stranded in the Canary Islands (2006–2012). A) Cervico-occipital fracture (animal no. 142; *Physeter macrocephalus*). There is a single, well-demarcated, deep incisive cut through the dorsal occipital region and the atlas vertebra. B) Cervico-occipital fracture (animal no. 142; *P*. *macrocephalus*). Multiple bone fracture surfaces are stained with blue antifouling paint from the vessel cutting edges (presumably the keel). C) Fat embolism (animal no. 86; *Kogia breviceps*). The microvasculature of the lung parenchyma, mainly alveolar capillaries, contains multiple osmium-tetroxyde-positive (black) fat emboli. OsO₄ (postfixation technique) and H&E. Inset (animal no. 86; *K*. *breviceps*): fat embolus obliterates and expands the vascular lumen. OsO₄ (postfixation technique) and H&E. D) Pulmonary bone emboli (animal no. 60; *K*. *breviceps*). There are multiple osseous emboli in the pulmonary microvasculature. H&E. Inset: Embolic osseous fragments consists of lamellar (mature) bone with multiple presumably viable osteocytes in lacunae. H&E.

VC involved 24/208 (11.5%) animals, representing 8 species (Table 1). The main morphologic diagnoses in this category are recorded in S10 Table. The etiologic diagnosis in these animals largely consisted of trauma, sharp or blunt, and was typically severe. Main findings included: cutaneous lacerations with muscle tearing; linear body sectioning of varying depth with exposure of viscera (thoracic, abdominal) and eventration (animal no. 15, 32, 74, 103, 104, 142, 150 and 216); single and multiple costal soft and bone tissue loss; evisceration (animal no. 3 and 200); cranioencephalic trauma (animal no. 10); and partial (animal no. 215) or complete (amputation) sectioning of the vertebral column (Fig 7a and 7b). Pulmonary and/or systemic fat embolism (Fig 7c) was observed in animal no. 10, 32, 74, 124 and 142. Animal no. 60 had multiple intravascular pulmonary osseous emboli (Fig 7d). Other findings included: hemothorax, hemoabdomen; and left pneumothorax with focal coronary arterial thrombosis (animal no. 200). Some animals had relevant coexisting diseases: animal no. 212 had multifocal pulmonary thromboembolism with intralesional *Crassicauda* sp. nematodes, chronic mesenteric arteritis and granulomatous nephritis with intralesional *Crassicauda* sp. nematodes; and a calf sperm whale (animal no. 215) had mycotic pneumonia.

### 3. Active stranding pathology

**Fig 8:**
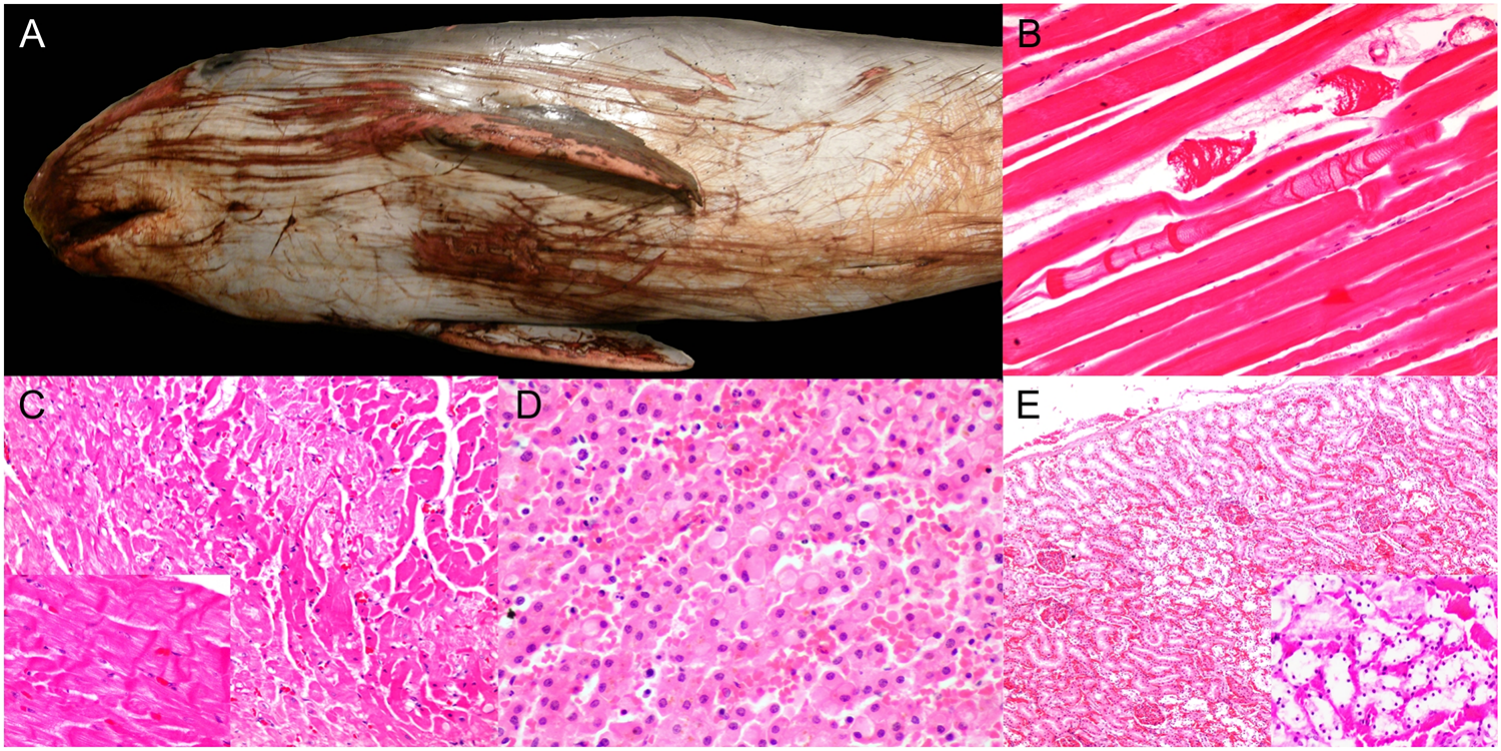
Panel of pathologic findings associated with active stranding in cetaceans stranded in the Canary Islands (2006–2012). A) Cutaneous erosions, lacerations and abrasions (animal no. 56; *Kogia breviceps*). There are multiple, longitudinal and somewhat parallel skin erosions, lacerations and abrasions with hemorrhage linked to live-stranding in a mildly abrupt substrate. B) Myodegeneration (animal no. 9; *Mesoplodon europaeus*). There are multifocal acutely degenerating myofibers showing variable patterns of segmental sarcoplasmic loss and edema. H&E. C) Cardiomyocyte degeneration (animal no. 56; *K*. *breviceps*). Multifocally, cardiomyocytes are swollen, hypercontracted and hypereosinophilic or pale and vacuolated. Inset: cardiomyocyte contraction band necrosis. H&E. D) Hepatocytic globules (animal no. 24; *Globicephala macrorhynchus*). Hepatocytes are multifocally expanded by intracytoplasmic, lightly eosinophilic single vacuoles with occasional pink points or strands. H&E. E) Tubulonephrosis (animal no. 138; *Grampus griseus*). Multifocally, the renal cortex has foci of pallor and tubular disruption. H&E. Inset: Multiple proximal convoluted tubules have degenerating, necrotic and sloughed epithelial cells and there is marked congestion. H&E.

A total of 35/224 (15.6%) animals stranded alive, representing 11 species (Table 1). Gross lesions suggestive of live-stranding were: superficial well-demarcated or irregular linear cutaneous erosions and lacerations of variable extent, mainly in the rostrum, the ventral aspect of the head, neck, thorax, abdomen, flanks and pectoral fins and tail fluke (Fig 8a); hemorrhage associated with myodegeneration, mainly in the *rectus abdominis*; multifocal pale foci in the myocardium; and systemic congestion, edema and petechiae in the brain, lungs, adrenal glands, liver, kidney and gastrointestinal tract. Classic microscopic findings of varying severity and extent were identified: mild to marked, multifocal, acute monophasic segmental skeletal (Fig 8b) and cardiac myodegeneration with contraction band necrosis, vacuolation and edema (Fig 8c); cytoplasmic eosinophilic globules within hepatocytes and ‘pink points’ (comprising acute phase proteins; Fig 8d) [34]; systemic and subendocardial and myocardial hemorrhage along with generalized congestion; acute adrenocortical degeneration and necrosis; and acute renal tubular degeneration and necrosis (Fig 8e) with or without myoglobin casts. The above findings often featured pre-existing conditions that were generally thought as the cause of stranding; CNS inflammation predominated in these cases (51.4%; 18/35).

## Discussion

Long-term pathology-based health monitoring of cetacean populations involves arduous, complex and demanding research and logistics. These pathologic investigations have obvious biases and limitations, *e*.*g*., the examined animals are not fully representative of the population, advanced decomposition of corpses at necropsy [35], the gain of scientific knowledge and associated outcomes exceed and justify the troubles. Furthermore, since the determination of cause of death often relies, to a certain extent, on the professional judgment of the supervising pathologist (from field necropsy to final report issuing in best case scenarios), interstudy variations in the criteria to assign causes of mortality are likely to occur [35].

### Stranding epidemiology

From January 2006 to December 2012, 320 cetaceans including 22 species stranded along the coastline of the Canary Islands. Postmortem examinations were performed on 70% of the animals, including 21 species (four mysticetes and 17 odontocetes). A previous study in the area (1999–2005) recorded 19 stranded cetacean species [12]. The variety of species recorded in both studies was similar, however, the common minke whale (*Balaenoptera acutorostrata*), Sowerby’s beaked whale (*Mesoplodon bidens*), harbor porpoise (*Phocoena phocoena*), and false killer whale were not recorded in the previous study. By contrast, the current study did not include Blainville’s beaked whale (*M*. *densirostris*) and spinner dolphin (*Stenella longirostris*) [12]. A cause for such subtle species differences is not apparent; further evaluation is precluded by the small number of individuals for those species.

Compared to 1999–2005 period [12], in the present study, the sex distribution of animals studied was more homogeneous (58 females, 79 males, 1 undetermined vs 105 females, 109 males, 10 undetermined); no sex-biased stranding tendency was apparent. Regarding age, we observed that mortality by species (*e*.*g*., short-beaked common dolphin, sperm whale [*Physeter macrocephalus*], pygmy sperm whale, striped dolphin, and Atlantic spotted dolphin) was high in young animals, decreasing in mature ones and finally increasing in aged ones [12,36,37]. In agreement with previous observations [12], stranding events occurred most often along the eastern coasts of the oriental islands. The yearly average of strandings (n = 45.7) during 2006–2012 was slightly higher than the period 1999–2005 (n = 39) yet peak months (March, April and May) were identical to those previously reported [12]. This small difference is likely not statistically significant.

The most probable CD was identified or inferred in 98.9% (91/92) ‘fresh’ and ‘very fresh’, 96.7% (59/61) ‘moderate autolysis’ and 81.7% (59/71) ‘advanced’ ‘very advanced autolysis’ carcasses. These results attested to the value of postmortem examinations in all animals regardless their decomposition status [12]; however, cautious interpretation of findings is mandatory in these cases to avoid overinterpretations.

### 1. Natural pathologic categories

#### 1.1 Pathology associated with good nutritional status

Etiologic diagnoses in NPGNS cases were: infectious (60%), parasitic (28.6%), other (*e*.*g*., gas encephalopathy, idiopathic hemorrhage, uterine rupture; 5.7%); gas embolism (2.8%), neoplasia (1.4%) and senile changes (1.4%). Our findings are somewhat similar to those previously observed in the area [12]. CeMV infection was confirmed in six animals; microscopic findings in these animals were in agreement with previous reports [38,39,40,41]. One of these animals had concomitant septicemia by *S*. *phocae* [26]. Lymphocytic meningoencephalitis in any odontocete species worldwide, particularly striped and bottlenose dolphins should prompt CeMV investigation [42]. Although IHC and PCR were not simultaneously performed with histologic examinations in a previous study in the Canary archipelago [12], retrospective IHC and PCR investigations confirmed CeMV infection in 36.8% previous ‘unknown origin meningoencephalitis’ cases [19]. Further studies to identify potential etiologic agents in cases of undetermined meningoencephalitis are warranted.

*E*. *rhusiopathiae* was isolated in an Atlantic bottlenose dolphin and an Atlantic spotted dolphin [17]. In both animals, lesions were suggestive of an acute septicemia [43,44,45,46,47]. Primary fungal meningoencephalitis by *A*. *fumigatus* was diagnosed in a striped dolphin calf. CNS infection by *Aspergillus* sp. has been recognized in a northern bottlenose whale (*Hyperoodon ampullatus*) [48], a harbor porpoise [49], three striped dolphins and a bottlenose dolphin co-infected by CeMV [38,50]. In this case, there was no evidence of systemic fungal infection and concomitant disease processes. CeMV and HV PCR analysis were negative. Four striped dolphins had toxoplasmosis agreeing with high prevalences for this species in the area [12]. The pathologic findings observed were similar to encephalitic and systemic presentations reported in different hosts and geographic locations [51].

Severe arterial and renal crassicaudiasis was observed in five CBW in this group [22]. *Crassicauda magna* was identified in two of them. Severe arterial lesions could result in fatalities from disseminated intravascular coagulation, multisystemic failure and severe renal damage, or major aggravation of coexisting disease [22]. Severe pterygoid sinusitis by *Nasitrema* sp. with middle and inner ear involvement and extension to the brain was observed in three common bottlenose dolphins and one short-beaked common dolphin. Gross and histologic findings were in agreement with previous reports [52,53,54,55,56,57,58,59]. An adult female striped dolphin had a large hepatic abscess firmly adhered to the pyloric compartment that ruptured and led to septic peritonitis. This process was associated with severe parasitism by *Brachycladium atlanticum* and *P*. *gastrophilus*. To the authors’ knowledge, this is a novel fatal outcome of severe digestive parasitism in cetaceans. Severe *Bolbosoma* sp. intestinal parasitism likely led to death in an adult female sei whale and a false killer whale. Different acanthocephalan species of genus *Corynosoma* and *Bolbosoma* parasitize the intestine of many odontocete and mysticete cetaceans [21,60,61,62,63]. All acanthocephalans are potentially pathogenic, as a result of their proboscids that penetrates the intestinal wall and may lead to peritonitis and severe disease [64]. *Bolbosoma capitatum* has been involved in mortalities of false killer whales due to intestinal obstruction, hemorrhagic enteritis, serositis (peritonitis) and severe anemia [65].

Severe *Crassicauda* sp. parasitization affecting the cervical and thoracic subcutis, fascia, and muscle, the cervical gland, and the rete mirabile was seen in an adult female pigmy sperm whale. These findings supported previous observations in which *C*. *magna* was identified in the cervico-thoracic subcutis and vascular plexuses that supply the CNS and other thoracic organs [66,67]. We believe severe *Crassicauda* sp. parasitization of the rete mirabile may contribute to severe vascular dysfunction. Severe urethral crassicaudiasis was diagnosed in an Atlantic spotted dolphin, recapitulating findings observed in baleen whales with urethral *C*. *boopis* infestation [68,69,70] and *Crassicauda* spp. in the lower urinary tract of other cetacean species, leading to partial or complete luminal obstruction [71,72].

Two animals presented lesions compatible with ‘gas embolism’ [73,74]. Two similar cases involving old Cuvier’s beaked whales with less pronounced hemorrhages were previously identified in the Canary archipelago [12]. Although gas embolism was linked to stranded ziphiids during military maneuvers and predation-related outcomes [28,73]; it is likely that gas embolism may be triggered by other non-sound-related sources, and individual factors such as age, health status and behavior, may be associated with this condition [28].

A glioblastoma multiforme was observed in an adult male Atlantic spotted dolphin [29]. CNS neoplasia is rare in cetaceans [75,76,77,78,79,80,81]. A live-stranded adult male pigmy sperm whale had CNS pathology suggestive of spongy myelinopathy with features resembling those observed in ammonia toxicity, endogenous metabolic toxicity (hepatic and renal encephalopathy), mitochondrial and idiopathic encephalopathies [82]. Nonetheless, the etiology in this case remains unknown.

Other uncommon causes of stranding and death were observed in this group. Idiopathic uterine rupture leading to hemoperitoneum and hypovolemic shock was seen in an adult female short-beaked common dolphin. In this case, the presence of a large post-gestational corpus albicans and marked dilatation of the ruptured uterine horn, suggested peri- or post-partum rupture. Uterine rupture was observed in an adult female Risso’s dolphin with an intraabdominal mummified fetus [83]. Focal intestinal torsion with strangulation and venous infarction led to fibrinous peritonitis and subsequent septic shock in an adult male Atlantic spotted dolphin, recapitulating features in previous reports [84]. Although uncertain, intestinal parasitism by *Bolbosoma* sp. might have been a predisposing factor in this case.

#### 1.2 Pathology associated with significant loss of nutritional status

Etiologic diagnoses were infectious (69.4%), parasitic (26.5%), senile changes (2%) and neoplasia (2%). Most individuals (29/33, 87.9%) with evidence of infectious disease had high endo- and ectoparasitic burdens. Our results suggest an overall higher number of deaths associated with infectious diseases (39/138; 28% vs 75/224, 33.5%), and lower age-related (14/138, 10.1% vs 2/224, 0.9%) morbidity and mortality than a previous study in the area [12]. Comparison with other small or large-scale pathology-based studies is somewhat troublesome, since a standardized etiopathological classification scheme was not followed. For instance, some studies reported only bacterial infections [85], and others considered viral, bacterial, fungal and parasitic processes altogether [4]. Nonetheless, when most studies from other geographical areas are scrutinized our data show similar or higher prevalence of infectious disease [4,12,79,86,87,88,89,90,91,92,93,94,95,96,97,98,99,100,101,102,103,104,105,106,107,108,109].

CeMV was detected by IHC and/or PCR in 10 NPSLNS cases representing five species. The chronic systemic and the chronic CNS-localized presentations predominated among animals in this group [42]. No characteristic eosinophilic intranuclear and intracytoplasmic inclusion bodies were observed in these animals. Herpesviral encephalitis was diagnosed in an adult male striped dolphin. Additionally, *Mycoplasma* sp. was isolated from chronic atlanto-occipital osteoarthritis in this animal. This case represents the first coinfection by *Herpesvirus* and *Mycoplasma* sp.in a cetacean species. Furthermore, about 30.6% (15/49) of NPSLNS cases had some degree of CNS inflammation of unknown etiology.

A juvenile female rough-toothed dolphin presented pathologic findings suggestive of upper digestive herpesviral disease along with bacterial septicemia, involving *Aeromonas* sp. and *Pseudomonas* sp. Fatal herpesviral systemic infections have been documented in several cetacean species, generally involving α-herpesvirus [110,111,112,113,114]. The individual pathogenic role of each bacteria is uncertain yet both genera have been linked to disease in cetaceans [2,106,115]. *W*. *chitiniclastica* septicemia was diagnosed in an adult male short-beaked common dolphin. This was the first description for this emerging human pathogen in a marine mammal species [30]. This agent had been linked to fatal septicemia in two human patients [116,117] and a white-tailed deer (*Odocoileus virginianus*) [118]. *P*. *aeruginosa* was associated with severe nephrolithiasis and ureterolithiasis with unilateral hydronephrosis and hydroureter, suppurative pyelonephritis and renal infarcts in an adult male short-beaked common dolphin. Additional histopathologic evidence (*i*.*e*., multiorgan leukocytosis, meningitis with intravascular bacteria, adrenalitis and mesenteric lymph node sinus histiocytosis with bacteria) supported bacterial septicemia; however, no bacteria were grown from liver, lung and brain tissue. *P*. *aeruginosa* has been previously linked to pyelonephritis [119] and as a primary pathogen in lower urinary tract infections [120]. Moreover, several species of *Pseudomonas* have been implicated in localized and systemic infections in cetaceans [2,106]. In the present case, *P*. *aeruginosa* infection could have played a role in chronic renal insufficiency and could explain additional systemic findings.

We observed other NPSLNS cases characterized by systemic infectious inflammatory disease of unknown etiology involving peritonitis in an adult male pigmy sperm whale, an adult female and a male striped dolphin. In the first, a hepatic abscess was associated with peritonitis and hemoabdomen. Hepatic changes included multiple infarcts, thrombosis and hepatocellular carcinoma. The later was likely incidental. The only other report of this neoplasm in a cetacean involved beluga whales [121]. A bacterial cause for hepatic abscesses is suspected albeit it remains unknown. In the second case, a perforating ulcer in the keratinized compartment led to septic peritonitis; however, its etiology remains unknown. Infectious or not, areas of gastric erosion and ulceration may perforate and lead to peritonitis [103,122,123,124]. The third case had fibrinosuppurative peritonitis, pyothorax, pleuropneumonia and pericarditis with intralesional bacteria. Compelling evidences support a bacterial septicemia; however, etiologic confirmation was not attained because no bacterial culture was performed in these cases.

Disseminated toxoplasmosis was determined in an Atlantic spotted dolphin calf. Gross and histologic findings were similar to those reported in many other cetacean species [51]. In this case, transplacental infection was highly suspected [125]. Risk factors for toxoplasmosis in cetaceans may include coinfection with CeMV [126], a low genetic diversity [127] and a coastal habitat [128]. Other potentially fatal parasitosis in this group involved severe *Nasitrema* sp. pterygoid sinusitis and otitis media and otitis interna with brain invasion [52,53,54,55,56,57,58,59]. *Nasitrema delphini* [31] was identified in one of the animals. We believe the severity and extent of these lesions could explain the stranding and death in both individuals. Systemic ciliated protozoosis was determined in an adult male rough-toothed dolphin, recapitulating lesions similar to previous reports [12,71,129,130,131]. Descriptions of ciliated protozoa in cetaceans include *Haematophagus megapterae* in humpback whales, fin whales and blue whales; *Kyaroikeus cetarius*, *K*. *cetarius*-like, *Planilamina ovata*, *P*. *magna*, *Balantidium* spp., and unidentified ciliates in mucosae and feces of several odontocetes and mysticetes worldwide [51]. These microorganisms are considered opportunistic, often concomitant in CeMV-infected hosts, and their pathogenicity is largely unknown. Systemic spread is not uncommon [51]. In the present case, PCR analysis for CeMV was negative. Additionally, a juvenile male short-finned pilot whale also had localized cutaneous and oral ciliate protozoal infestation. Parasitism as a cause of stranding and/or death has been widely debated [107,129,132,133]. This study corroborates the high occurrence of certain endoparasitoses and provides compelling evidence on the lethal potential for arterial and renal crassicaudiasis, pterygoid and CNS nasitremiasis, CNS and/or disseminated toxoplasmosis, hepatic brachycladiasis and urethral crassicaudiasis, particularly when vital organs, *e*.*g*., CNS, liver, kidneys, are affected.

A primary uterine T-cell lymphoma with disseminated metastases was diagnosed in an Atlantic spotted dolphin [32]. This case adds to the relatively common myeloid and lymphoid neoplasms in cetaceans [81,134,135,136,137,138,139].

#### 1.3 Neonatal/Perinatal pathology

NPP in cetaceans may include: abortion, dystocia, early maternal-filial separation or maternal neglect, early fatal intra- and interspecific interactions, infections, loss of passive transfer immunity, prematurity, and congenital malformations, among others [12,86]. Disturbances during gestation, labor, lactation and early behavioral abnormalities may be also included in this category [12,86]. We observed evidences of fetal distress (46%), dystocia (15%), infectious (15%), abortion (7.7%), congenital malformation (7.7%) and neonatal maternal-filial separation/maternal neglect (7.7%). Somewhat similar findings were found in a previous study in the area, predominantly involving ziphiids [12]. By contrast, this apparent predisposition of ziphiids was not observed in the present work.

A major neonatal/gestational pathologic condition in common bottlenose dolphins is prematurity [140]. The main risk factors for prematurity include: premature rupture of placental membranes; intrauterine infection; uterine, cervical and placental structural abnormalities; and multiple gestation. Our results suggest that fetal distress, based on compatible gross and histologic findings (e.g., pulmonary edema, aspirated squames and/or meconium, hyaline membranes) occurs in this category, regardless the primary underlying cause. Thus, perinatal respiratory distress or asphyxia is a common pathogenic mechanism in NPP-fatalities in cetaceans [86]. We also observed a case of presumed dystocia and of presumed maternal-filial separation. Distinguishing the former from a potential non-lethal dystocic or non-dystocic birth followed by neonatal weakness, traumatic interaction and posterior maternal-filial separation may prove extremely difficult; however, distribution of congestive and hemorrhagic areas, particularly circumferential ones potentially related to uterine contraction or birth canal pressure may be suggestive. Furthermore, several animals in this study presented evidence of neonatal/perinatal infection: a presumptively premature short-finned pilot whale with sepsis and fetal distress; and a calf pigmy sperm whale with omphaloarteritis and phlebitis, and evidence of sepsis. In both cases, the etiologic agent remains unknown. Noteworthy, a presumably premature striped dolphin presented segmental intestinal atresia, a spinal meningocele and incomplete neurocranium development. Intestinal atresia bore similarity with jejuno-ileal atresia described in humans, the most common form of intestinal atresia [141]. Meningocele is the most simple ‘open neural tube’ type defect, and is characterized by cystic meningeal dilatation containing cerebrospinal fluid without neural tissue [142]. These developmental malformations have not been reported to date in cetacean species.

#### 1.4 Intra- and interspecific traumatic interactions

Intra- and interspecific interactions among cetaceans are diverse and complex, and often aggressive in nature [143,144,145,146,147]. Several reports describe severe injuries in calves, including infanticide [145,146,148,149,150]. Predation of other cetaceans is well known for the killer whale (*Orcinus orca*), which has been observed attacking or harassing over 20 different species of cetaceans, including families Balaenopteridae, Balaenidae, Eschrichtiidae, Physeteridae, Ziphiidae, Monodontidae, Delphinidae and Phocoenidae [151,152,153,154,155,156,157,158,159].

We detected higher fatal intra-/interspecific interactions than a previous study in the area (17.8% vs 4.3%), involving a wider range of species (13vs 5) [12]. We found short-finned pilot whales were most prevalent, which is in agreement with previous studies in other geographic areas, often involving young adult males and linked to reproductive initiation [160]. Consistent trauma-associated pathologic findings in this category were associated with cutaneous tooth rake marking [161], external and internal hemorrhages and bone fractures [12]. Less often, pulmonary fat emboli and myo/hemoglobinuric tubulonephrosis were observed. Evidence of traumatic events and concomitant infectious inflammatory disease, largely involving the CNS or systemic disease was observed in 12/37 (32.4%) animals. Noteworthy, an unusual fatal traumatic event involved a juvenile male false killer whale in poor body condition and a stingray’s spine perforating the tongue. Stingray spine injury was considered a potential cause of stranding or beaching in killer whales [162]. Another unusual case involved a male adult Risso’s dolphin with systemic gas embolism likely associated with complicated predation on squids [28].

### 2. Anthropogenic pathologic categories

#### 2.1 Interaction with fishing activities

Bycatch is the greatest anthropogenic threat to cetaceans; gillnets, trawl nets, trammel nets, purse seine nets and longlines pose the greatest risks for these species worldwide [163]. Often, small whales, dolphins and porpoises trapped in fishing nets die by asphyxia; conversely, large whales may be able to release themselves or continue entangled during long periods leading to chronic debilitating lesions and/or eventual death [164]. Very few studies have successfully evaluated and quantified real fishing impact on cetacean populations [165,166]; thus, bycatch is largely believed to be under-reported. A considerable number of publications have reviewed the gross, and to a lesser extent, the histologic findings associated with bycatch in cetaceans [105,167,168,169,170]. The greater the number of indicator criteria identified in an individual case, the greater the likelihood of an accurate diagnosis of bycatch [170].

We detected a lower prevalence of fatal IFA than a previous study in the area [12]. In these animals, we observed lesions associated with contact with fishing nets, entanglement, lacerations, as well as craneoencephalic contusions, hematomas and bone fractures, analogous to previous reports [12,168]. Other findings in this category were deep linear cutaneous wounds along with thoracic wall and lung perforation that lead to hemothorax, and necrotizing osteomyelitis due to perforating hook in the mandible of an Atlantic spotted dolphin. Additionally, we detected 4/10 (40%) animals with moderate to severe underlying inflammatory processes including CNS inflammation and verminous pneumonia. Despite the presence of algae, diatoms, dinoflagellates, foraminifera and mineral grains as a common feature in bycaught harbor porpoises due to terminal aspiration of water [171,172], we did not observe such marine elements in any of the animals of this group by histologic examination.

#### 2.2 Foreign body-associated pathology

Marine debris are recognized as a major worldwide environmental, economic, public health and esthetic issue, posing a complex and multidimensional challenge with important consequences for the marine environment and human activity [173,174]. Between 40–80% of marine debris derive from plastics, mostly originating on land, although lost or discarded fishing nets might represent a considerable proportion, especially along the continental shelves and remote islands [175]. Excellent review articles on this issue have been published [176,177,178,179]. For marine fauna, particularly cetaceans, major impacts linked to marine debris include ingestion and entanglements [180]. Ingestion may cause obstruction of the digestive tract leading to inanition, while entanglement may result in drowning, asphyxiation, or strangulation [178]. Sublethal effects are also recognized: chronic ingestion and entanglement may compromise feeding and digestion capacity, resulting in malnutrition, disease, and reduction in reproductive performance, growing rates and longevity [181,182]. In addition, they may transfer persistent organic pollutants among others, phenomenon especially concerning for microplastics [183]. Predisposing factors for foreign body ingestion in cetaceans remain obscure, and several hypotheses have been formulated [184,185].

In this study, we found a lower prevalence of FBAP as a CD than a previous study in the area (6/138, 4.3% vs 5/224, 2.2%). [12]. The main conditions were: gastric obstruction and impaction, followed by gastric perforation and prolonged entanglement. Gastric impactions were mainly associated with abundant plastics and the single case of gastric perforation was secondary to coiled wire, leading to septic fibrinosuppurative peritonitis and death. A common minke whale calf had severe cachexia associated with prolonged entanglement linked to a presumably fishing net adrift, which had led to severe constrictive maxillary and mandibular disease. Lesions inflicted by fishing nets adrift are analogous to those of bycatch, and sometimes discerning between the two situations is not possible. In these cases, progressive constriction and tissue damage of any component of the alimentary tract and musculoskeletal system could preclude foraging/feeding behavior and locomotion, resulting in inanition and death [186,187]. Species with apparent predisposition to foreign body ingestion include: sperm whales [188,189], beaked whales [185,189,190,191,192], Franciscana (*Pontoporia blainvillei*)[193], and kogiids [185,194,195].

#### 2.3 Vessel collisions

Ship collision is a major threat to cetaceans worldwide [196,197,198,199]. A wide range of vessels have been reported to have struck whales yet most severe and lethal lesions are typically inflicted by large vessels (≥80 m-length) and by those sailing at speeds greater than 14 knots [200]. Vessel collisions with cetaceans have been widely described in cetaceans in the Canary archipelago [12,201,202,203] over the last decades, aggravated by increased maritime traffic, the increased size and speed of the vessels, and the convergence of some of the main sailing routes with areas where cetaceans congregate [12].

VC-associated lesions in cetaceans are broadly divided into two categories: 1) sharp trauma, *i*.*e*., thrust, incise and cutting, often as a result of direct contact with propeller edges and rudder; and 2) blunt trauma, *i*.*e*., abrasions, lacerations, contusions and skeletal fractures [164]. This study found a higher prevalence of fatal VC than a previous study in the area (24/224, 10.7% vs 8/138, 5.8%) [12], involving eight species, mostly deep and long-lasting divers. In our study, sperm whales were the most frequently affected, agreeing with previous observations in the area [12,201]. Long periods of socialization and surface breaks after prolonged dives would be risk factors in this species [201]. In the present study, eight sperm whales were calves, two juveniles and one adult, denoting a clear predisposition of the youngest individuals [12]. This age-biased predisposition is probably related to larger times the offspring spend on the surface compared to adults, and a possible ability acquired by adults to avoid collisions [200]. Likewise, argued risk factors for deep and prolonged diving species would be long surface rests with relatively slow swimming [12,200]. Other species appear at higher risk worldwide: fin whale (*Balaenoptera physalus*), the northern right whales (*Eubalaena glacialis*), southern right whales (*Eubalaena australis*), humpback whales and gray whales (*Eschrichtius robustus*) [200].

VC-diagnostic criteria have been revised [164,204]. Briefly, these would include one or more cuts, demonstrated ante-mortem bone fractures, hematoma(s) and/or hemorrhage(s). Microscopically, typical findings of acute trauma may include edema and subcutaneous hemorrhage, and hemorrhage with myodegeneration, necrosis, and muscle contracture underlying the collision site. A recent study further characterized microscopic lesions in axial muscles in VC cases [205]. In the present study, all animals with VC diagnosis and microscopic examination of grossly injured or adjacent muscle had acute myodegenerative processes analogous to above studies [164,204,205]. The occurrence of non-fatal and fatal VC could be underestimated because some collisions only inflict blunt trauma that could go unnoticed in postmortem examinations and because some whales struck by vessels may sink and go unnoticed to scientists or official authorities [164,206]. Nonetheless, they may also have been overestimated due to incorrect interpretation of collisions with floating dead whales; determination of fatty, cartilaginous and/or bone embolism may aid to achieve a more accurate diagnosis in these cases. Our findings further attest to the importance of VC in cetaceans of the Canary archipelago [12,201,202,203].

#### 2.4. Active stranding pathology

Regardless of whether or not the animal is seriously ill, active stranding by itself involves an anomalous and extreme situation for an organism that is not anatomically and physiologically adapted to a solid surface, or to environmental conditions different from those of the aquatic environment [207,208]. Live-stranded cetaceans are often weakened at the time of rescue, their condition deteriorates during the capture and restraint, and they may die after a period in captivity [2]. In cetaceans, acute deaths after stranding may be attributed to hyperthermia and catecholaminergic and neurogenic shock (so called ‘stress response syndrome’ or ‘alarm reaction’), and multiorgan failure, *e*.*g*., renal dysfunction. This is typically referred to and thought to be analogous to capture myopathy in terrestrial mammals, particularly in prolonged rescues and rehabilitation efforts [13,24,25,124].

Although macroscopic lesions are often not very evident, histopathologic findings in capture myopathy may include areas of necrosis in the CNS, lung, liver, intestine, pancreas and lymph nodes; acute skeletal and cardiac myodegeneration; and tubular nephrosis often with intratubular myoglobin casts [23,24,25]. Furthermore, serum creatinine phosphokinase, aspartate aminotransferase and alanine aminotransferase may be elevated [209,210,211]. Presumably, the muscle damage is a direct consequence of stranding and not the cause, reflecting stress, overexertion, trauma, and crush injury [25,209,210]. In the present study, nearly 16% of the animals were confirmed to live-strand, lower than previous observations in the area (37/138, 26.8%) [12]. All these animals had lesions of varying severity and extent, compatible with ‘active stranding pathology’ and major involvement of the integumentary, musculoskeletal, cardiovascular and urinary systems. The above lesions generally overlapped and sometimes were masked by underlying pathologies, presumably responsible (or not) for active stranding [23,24,25,212]. These were of diverse nature yet CNS inflammation was most frequent (18/35, 51.4%) [12,24]. Despite the high incidence of active stranding-associated lesions, the pathogenetic mechanisms in these species are not fully resolved. Major players would include local to generalized vasospasm (catecholamines, neurogenic), local to generalized vasodilation (shock, impeded venous flow return by body compression), direct traumatic injury of muscle and viscera, and reperfusion damage. The latter particularly deserves further research in cetaceans.

### Conclusion

Herein, our results suggest that direct human activity was responsible for 19% of cetaceans’ deaths, while natural pathologic categories would account for 81% in the Canary archipelago between 2006 and 2012. Within natural PCs, those associated with good nutritional status represented 33.6%, whereas, those associated with significant loss of nutritional status represented 23.5%. Fatal intra- and interespecific traumatic interactions were 17.8%. Vessel collisions included 11.5%. Neonatal/perinatal pathology involved 6.2%. Fatal interaction with fishing activities comprised 4.8%. Within anthropogenic PCs, foreign body-associated pathology represented 2.4%. A CD could not be determined in 7.7% cases. Natural PCs were dominated by infectious and parasitic disease processes. These results, integrating novel findings and published reports, aid in delineating baseline knowledge on cetacean pathology and may be of value to rehabilitators, caregivers, diagnosticians and future conservation policies.

## Supporting information

S1 Table Biological and epidemiological data of 224 stranded and necropsied cetaceans. Sex: female (F), male (M). Age: fetus (F), neonate (N), calf (C), juvenile (Jv), subadult (Sad), adult (Ad). Stranding date (SD; mm/dd/yy). Type of stranding (TS). Stranding location, island (IS): Gran Canaria (GC), Fuerteventura (FT), Lanzarote (LZ), Tenerife (TF), La Gomera (LG), El Hierro (EH), La Palma (LP), La Graciosa (LGra). Conservation status (CS): Very fresh (VF), fresh (F), moderate autolysis (MA), advanced autolysis (AA), very advanced autolysis (VAA).
(DOCX)

S2 Table Details of immunohistochemical analyses performed on formalin-fixed, paraffin-embedded tissue sections from selected cetacean species, including primary antibody (Ab), manufacturer, clonality, dilution, pretreatment, incubation, secondary Ab, manufacturer, and visualization system. CD: Cluster of differentiation; CK: cytokeratin; GFAP: glial fibrillary acid protein, NSE: neuron specific enolase, MAC387: myeloid/histiocytic antigen; CDV: canine distemper virus; HSV1: Herpes Simplex virus 1: HIER: Heat-induced epitope retrieval; ABC: Avidin-Biotin-Peroxidase complex.
(DOCX)

S3 Table Tissues submitted for microbiological analysis and results from a subset of 224 stranded and necropsied cetaceans. (DOCX)

S4 Table Main morphologic and etiologic diagnoses in animals included in ‘pathology associated with good nutritional status’. (DOCX)

S5 Table Main morphologic and etiologic diagnoses in animals included in ‘natural pathology associated with significant loss of nutritional status’. (DOCX)

S6 Table Main morphologic and etiologic diagnoses in animals included in ‘neonatal and perinatal pathology’. (DOCX)

S7 Table Main morphologic and etiologic diagnoses in animals included in ‘intra- and interspecific traumatic interactions’. (DOCX)

S8 Table Main morphologic and etiologic diagnoses in animals included in ‘interaction with fishing activities’. (DOCX)

S9 Table Main morphologic and etiologic diagnoses in animals included in ‘foreign body-associated pathology’. (DOCX)

S10 Table Main morphologic and etiologic diagnoses in animals included in ‘vessel collision’. (DOCX)
