# Supplementary material for: Pathologic findings and causes of death of stranded cetaceans in the Canary Islands (2006-2012)
Source: PLoS One. 2018 Oct 5;13(10):e0204444. doi: 10.1371/journal.pone.0204444 (PMC6173391; doi:10.1371/journal.pone.0204444)
Supplement: S1 Table — Sex: female (F), male (M). Age: fetus (F), neonate (N), calf (C), juvenile (Jv), subadult (Sad), adult (Ad). Stranding date (SD; mm/dd/yy). Type of stranding (TS). Stranding location, island (IS): Gran Canaria (GC), Fuerteventura (FT), Lanzarote (LZ), Tenerife (TF), La Gomera (LG), El Hierro (EH), La Palma (LP), La Graciosa (LGra). Conservation status (CS): Very fresh (VF), fresh (F), moderate autolysis (MA), advanced autolysis (AA), very advanced autolysis (VAA). (DOCX) [file pone.0204444.s001.docx]

**S1 Table. Biological and epidemiological data of 224 stranded and necropsied cetaceans**. Sex: female (F), male (M). Age: fetus (F), neonate (N), calf (C), juvenile (Jv), subadult (Sad), adult (Ad). Stranding date (SD; mm/dd/yy). Type of stranding (TS). Stranding location, island (IS): Gran Canaria (GC), Fuerteventura (FT), Lanzarote (LZ), Tenerife (TF), La Gomera (LG), El Hierro (EH), La Palma (LP), La Graciosa (LGra). Conservation status (CS): Very fresh (VF), fresh (F), moderate autolysis (MA), advanced autolysis (AA), very advanced autolysis (VAA).

| **No** | **Species** | **Sex** | **Age** | **SD** | **TS** | **IS** | **CS** |
| --- | --- | --- | --- | --- | --- | --- | --- |
| 1 | *Stenella frontalis* | M | Ad | 2/7/06 | D | GC | F |
| 2 | *Globicephala macrorhynchus* | F | Ad | 2/11/06 | D | GC | AA |
| 3 | *Kogia breviceps* | M | Jv | 2/19/06 | D | GC | VAA |
| 4 | *Stenella coeruleoalba* | M | Sad | 2/20/06 | D | FT | F |
| 5 | *Delphinus delphis* | F | Ad | 3/5/06 | D | GC | F |
| 6 | *Kogia breviceps* | M | Ad | 3/17/06 | D | LZ | MA |
| 7 | *Stenella frontalis* | M | Ad | 3/17/06 | D | LZ | AA |
| 8 | *Tursiops truncatus* | F | Ad | 3/24/06 | D | LZ | MA |
| 9 | *Mesoplodon europaeus* | F | Sad | 3/28/06 | Al | EH | MA |
| 10 | *Mesoplodon europaeus* | F | Sad | 3/28/06 | Al | EH | F |
| 11 | *Kogia breviceps* | F | Ad | 3/31/06 | D | LG | MA |
| 12 | *Physeter macrocephalus* | F | Sad | 4/1/06 | D | FT | AA |
| 13 | *Mesoplodon europaeus* | F | Jv | 4/6/06 | D | GC | F |
| 14 | *Globicephala macrorhynchus* | F | Ad | 4/9/06 | Al | FT | F |
| 15 | *Physeter macrocephalus* | F | C | 4/27/06 | D | TF | F |
| 16 | *Delphinus delphis* | M | Ad | 4/30/06 | D | TF | AA |
| 17 | *Globicephala macrorhynchus* | F | Jv | 5/11/06 | D | GC | F |
| 18 | *Kogia sima* | F | Ad | 5/23/06 | D | FT | F |
| 19 | *Ziphius cavirostris* | ND | Jv | 7/6/06 | D | TF | AA |
| 20 | *Mesoplodon europaeus* | M | C | 7/28/06 | D | TF | MA |
| 21 | *Stenella frontalis* | F | N | 7/28/06 | D | TF | MA |
| 22 | *Kogia breviceps* | F | Ad | 8/13/06 | D | FT | AA |
| 23 | *Phocoena phocoena* | M | C | 8/15/06 | D | FT | F |
| 24 | *Globicephala macrorhynchus* | M | C | 10/10/06 | Al | GC | VF |
| 25 | *Globicephala macrorhynchus* | ND | Ad | 11/30/06 | D | GC | VAA |
| 26 | *Stenella frontalis* | F | Ad | 12/20/06 | Al | TF | VF |
| 27 | *Stenella frontalis* | F | Sad | 1/1/07 | D | LZ | VF |
| 28 | *Delphinus delphis* | M | Sad | 1/20/07 | D | GC | MA |
| 29 | *Stenella coeruleoalba* | M | C | 2/4/07 | D | LZ | MA |
| 30 | *Stenella coeruleoalba* | M | C | 2/9/07 | D | GC | AA |
| 31 | *Stenella frontalis* | M | Jv | 2/20/07 | D | GC | MA |
| 32 | *Balaenoptera physalus* | F | Ad | 2/23/07 | D | GC | MA |
| 33 | *Stenella coeruleoalba* | M | Ad | 3/10/07 | Al | TF | F |
| 34 | *Stenella frontalis* | F | Ad | 3/12/07 | D | TF | F |
| 35 | *Balaenoptera borealis* | F | Sad | 3/20/07 | D | GC | AA |
| 36 | *Delphinus delphis* | F | Ad | 3/26/07 | Al | LGra | F |
| 37 | *Stenella coeruleoalba* | M | Ad | 4/3/07 | D | FT | F |
| 38 | *Stenella coeruleoalba* | M | N | 4/5/07 | D | TF | F |
| 39 | *Stenella coeruleoalba* | F | Ad | 4/6/07 | D | FT | F |
| 40 | *Kogia breviceps* | F | Ad | 4/6/07 | D | TF | MA |
| 41 | *Stenella frontalis* | M | Ad | 4/6/07 | Al | TF | VF |
| 42 | *Balaenoptera acutorostrata* | M | Ad | 4/16/07 | D | LZ | VF |
| 43 | *Stenella coeruleoalba* | M | Sad | 4/16/07 | D | TF | F |
| 44 | *Stenella coeruleoalba* | M | Sad | 4/23/07 | D | FT | MA |
| 45 | *Delphinus delphis* | M | Ad | 4/28/07 | D | TF | MA |
| 46 | *Stenella frontalis* | M | Ad | 5/9/07 | D | GC | MA |
| 47 | *Kogia breviceps* | ND | Ad | 6/20/07 | D | GC | VAA |
| 48 | *Globicephala macrorhynchus* | M | C | 7/3/07 | D | TF | MA |
| 49 | *Stenella frontalis* | F | Ad | 7/24/07 | D | FT | F |
| 50 | *Stenella frontalis* | M | Ad | 6/24/07 | D | FT | F |
| 51 | *Kogia breviceps* | F | Ad | 8/29/07 | D | LZ | MA |
| 52 | *Globicephala macrorhynchus* | M | N | 9/7/07 | D | TF | VAA |
| 53 | *Stenella coeruleoalba* | M | Ad | 9/25/07 | D | FT | F |
| 54 | *Stenella coeruleoalba* | F | Ad | 11/13/07 | D | FT | AA |
| 55 | *Tursiops truncatus* | M | Sad | 11/14/07 | D | TF | AA |
| 56 | *Kogia breviceps* | M | Ad | 12/2/07 | Al | GC | VF |
| 57 | *Globicephala macrorhynchus* | F | N | 12/26/07 | Al | GC | VF |
| 58 | *Delphinus delphis* | M | C | 1/14/08 | D | TF | F |
| 59 | *Tursiops truncatus* | F | Ad | 1/18/08 | D | TF | F |
| 60 | *Kogia breviceps* | F | Jv | 1/31/08 | D | TF | AA |
| 61 | *Stenella coeruleoalba* | F | Sad | 2/5/08 | D | FT | MA |
| 62 | *Delphinus delphis* | M | C | 3/8/08 | D | TF | MA |
| 63 | *Stenella coeruleoalba* | M | C | 3/10/08 | Al | GC | VF |
| 64 | *Pseudorca crassidens* | M | Jv | 3/11/08 | Al | LZ | F |
| 65 | *Stenella frontalis* | M | Ad | 3/14/08 | D | TF | MA |
| 66 | *Stenella frontalis* | F | Jv | 3/23/08 | D | TF | MA |
| 67 | *Stenella frontalis* | F | Ad | 3/26/08 | D | GC | F |
| 68 | *Steno bredanensis* | F | Jv | 3/28/08 | D | GC | F |
| 69 | *Tursiops truncatus* | F | Ad | 3/29/08 | D | GC | MA |
| 70 | *Stenella frontalis* | M | C | 3/31/08 | D | GC | F |
| 71 | *Delphinus delphis* | F | Ad | 4/5/08 | D | FT | AA |
| 72 | *Stenella frontalis* | M | Jv | 4/7/08 | D | LZ | AA |
| 73 | *Delphinus delphis* | F | Jv | 4/2/08 | D | TF | AA |
| 74 | *Physeter macrocephalus* | F | C | 4/10/08 | D | GC | AA |
| 75 | *Stenella coeruleoalba* | F | Jv | 4/12/08 | D | FT | F |
| 76 | *Steno bredanensis* | F | Sad | 4/14/08 | D | GC | AA |
| 77 | *Grampus griseus* | M | Jv | 4/20/08 | Al | TF | F |
| 78 | *Stenella frontalis* | F | Ad | 4/24/08 | D | GC | AA |
| 79 | *Stenella frontalis* | F | C | 4/26/08 | D | FT | MA |
| 80 | *Stenella frontalis* | F | C | 5/1/08 | D | FT | MA |
| 81 | *Tursiops truncatus* | F | Sad | 5/13/08 | D | TF | F |
| 82 | *Stenella frontalis* | ND | C | 5/15/08 | D | GC | AA |
| 83 | *Stenella frontalis* | M | C | 5/25/08 | D | GC | MA |
| 84 | *Grampus griseus* | F | Ad | 6/17/08 | Al | GC | F |
| 85 | *Tursiops truncatus* | F | Sad | 6/24/08 | D | TF | AA |
| 86 | *Kogia breviceps* | M | Ad | 6/27/08 | D | LZ | MA |
| 87 | *Stenella coeruleoalba* | M | C | 7/9/08 | D | FT | AA |
| 88 | *Stenella frontalis* | F | C | 7/20/08 | D | LZ | AA |
| 89 | *Physeter macrocephalus* | F | N | 7/22/08 | Al | LG | VF |
| 90 | *Globicephala macrorhynchus* | M | Jv | 8/9/08 | D | TF | MA |
| 91 | *Stenella coeruleoalba* | F | Ad | 10/26/08 | D | FT | F |
| 92 | *Ziphius cavirostris* | F | Sad | 11/6/08 | D | FT | F |
| 93 | *Grampus griseus* | F | Jv | 11/7/08 | D | FT | F |
| 94 | *Steno bredanensis* | M | Ad | 11/18/08 | Al | GC | F |
| 95 | *Stenella coeruleoalba* | M | Ad | 12/31/08 | D | LZ | F |
| 96 | *Stenella frontalis* | F | C | 1/10/09 | D | TF | MA |
| 97 | *Stenella coeruleoalba* | F | Ad | 1/22/09 | D | FT | F |
| 98 | *Stenella coeruleoalba* | F | C | 2/9/09 | D | GC | F |
| 99 | *Globicephala macrorhynchus* | ND | Ad | 2/18/09 | D | GC | AA |
| 100 | *Delphinus delphis* | F | Ad | 2/27/09 | D | LZ | AA |
| 101 | *Delphinus delphis* | F | Ad | 3/6/09 | D | TF | F |
| 102 | *Grampus griseus* | M | Ad | 3/6/09 | D | FT | F |
| 103 | *Physeter macrocephalus* | F | Ad | 3/13/09 | D | TF | F |
| 104 | *Physeter macrocephalus* | M | C | 3/13/09 | D | TF | MA |
| 105 | *Stenella coeruleoalba* | F | C | 4/2/09 | D | GC | AA |
| 106 | *Stenella coeruleoalba* | F | Ad | 4/6/09 | D | LZ | AA |
| 107 | *Mesoplodon bidens* | F | C | 4/9/09 | D | GC | F |
| 108 | *Stenella coeruleoalba* | M | C | 4/25/09 | D | GC | MA |
| 109 | *Globicephala macrorhynchus* | M | Ad | 4/29/09 | D | LZ | AA |
| 110 | *Stenella coeruleoalba* | M | Ad | 4/29/09 | D | GC | MA |
| 111 | *Steno bredanensis* | ND | Ad | 5/2/09 | D | GC | VAA |
| 112 | *Balaenoptera acutorostrata* | F | C | 5/8/09 | D | GC | AA |
| 113 | *Globicephala macrorhynchus* | M | Ad | 5/13/09 | D | FT | AA |
| 114 | *Stenella coeruleoalba* | M | Ad | 6/18/09 | D | TF | MA |
| 115 | *Physeter macrocephalus* | F | Ad | 6/27/09 | D | TF | MA |
| 116 | *Globicephala macrorhynchus* | M | Ad | 7/6/09 | D | TF | F |
| 117 | *Stenella coeruleoalba* | F | Sad | 7/22/09 | Al | LZ | F |
| 118 | *Ziphius cavirostris* | F | Ad | 9/21/09 | D | GC | AA |
| 119 | *Globicephala macrorhynchus* | M | Ad | 9/23/09 | D | GC | AA |
| 120 | *Tursiops truncatus* | M | Jv | 10/23/09 | D | TF | AA |
| 121 | *Stenella coeruleoalba* | F | N | 10/24/09 | D | GC | MA |
| 122 | *Stenella frontalis* | F | Ad | 11/2/09 | Al | GC | VF |
| 123 | *Tursiops truncatus* | M | Sad | 12/9/09 | D | TF | MA |
| 124 | *Mesoplodon europaeus* | M | Ad | 12/14/09 | D | LZ | F |
| 125 | *Globicephala macrorhynchus* | M | Ad | 1/9/10 | D | GC | F |
| 126 | *Stenella frontalis* | M | Ad | 2/26/10 | Al | FT | F |
| 127 | *Delphinus delphis* | M | Ad | 3/2/10 | D | GC | F |
| 128 | *Stenella coeruleoalba* | M | C | 3/14/10 | D | LZ | MA |
| 129 | *Physeter macrocephalus* | F | C | 3/16/10 | D | TF | MA |
| 130 | *Delphinus delphis* | ND | Jv | 3/23/10 | D | FT | AA |
| 131 | *Stenella frontalis* | M | Ad | 3/24/10 | D | LZ | MA |
| 132 | *Balaenoptera acutorostrata* | M | C | 3/24/10 | D | TF | F |
| 133 | *Tursiops truncatus* | F | Ad | 3/29/10 | D | TF | F |
| 134 | *Stenella coeruleoalba* | F | Ad | 3/29/10 | Al | FT | MA |
| 135 | *Stenella frontalis* | F | Ad | 4/13/10 | D | TF | F |
| 136 | *Stenella frontalis* | M | Jv | 4/18/10 | D | LG | F |
| 137 | *Grampus griseus* | M | Ad | 4/20/10 | D | FT | MA |
| 138 | *Grampus griseus* | M | Sad | 4/22/10 | Al | TF | VF |
| 139 | *Stenella coeruleoalba* | F | Ad | 5/3/10 | D | GC | F |
| 140 | *Kogia sima* | ND | Ad | 7/1/10 | D | GC | VAA |
| 141 | *Tursiops truncatus* | M | Ad | 7/2/10 | D | LZ | AA |
| 142 | *Physeter macrocephalus* | M | Jv | 7/9/10 | D | TF | MA |
| 143 | *Kogia breviceps* | M | N | 7/11/10 | D | FT | VAA |
| 144 | *Stenella coeruleoalba* | M | Ad | 7/17/10 | D | GC | F |
| 145 | *Mesoplodon europaeus* | M | Ad | 8/29/10 | D | FT | MA |
| 146 | *Stenella frontalis* | M | C | 9/16/10 | D | TF | F |
| 147 | *Grampus griseus* | F | Ad | 9/17/10 | D | TF | F |
| 148 | *Balaenoptera borealis* | M | Jv | 11/7/10 | D | GC | AA |
| 149 | *Stenella coeruleoalba* | F | Ad | 12/21/10 | Al | GC | F |
| 150 | *Physeter macrocephalus* | M | C | 1/29/11 | D | GC | AA |
| 151 | *Delphinus delphis* | M | Ad | 2/5/11 | D | FT | F |
| 152 | *Stenella coeruleoalba* | F | Ad | 2/10/11 | D | LZ | F |
| 153 | *Kogia breviceps* | M | Ad | 2/12/11 | D | FT | AA |
| 154 | *Lagenodelphis hosei* | M | Sad | 2/20/11 | D | FT | AA |
| 155 | *Tursiops truncatus* | F | Ad | 3/5/11 | D | TF | AA |
| 156 | *Stenella frontalis* | F | C | 3/6/11 | D | TF | AA |
| 157 | *Tursiops truncatus* | M | Ad | 3/22/11 | D | LZ | F |
| 158 | *Grampus griseus* | F | Ad | 3/22/11 | D | LZ | AA |
| 159 | *Stenella coeruleoalba* | F | Ad | 3/26/11 | D | TF | F |
| 160 | *Delphinus delphis* | ND | Sad | 3/11/11 | D | FT | AA |
| 161 | *Kogia breviceps* | M | C | 3/28/11 | D | FT | MA |
| 162 | *Delphinus delphis* | F | Ad | 3/29/11 | D | FT | AA |
| 163 | *Globicephala macrorhynchus* | F | Ad | 4/1/11 | D | GC | AA |
| 164 | *Physeter macrocephalus* | M | Jv | 4/26/11 | D | TF | MA |
| 165 | *Stenella coeruleoalba* | M | Ad | 5/1/11 | Al | GC | F |
| 166 | *Globicephala macrorhynchus* | M | C | 5/10/11 | D | FT | MA |
| 167 | *Ziphius cavirostris* | F | Ad | 5/16/11 | D | LZ | F |
| 168 | *Stenella coeruleoalba* | M | Ad | 5/16/11 | Al | TF | F |
| 169 | *Grampus griseus* | F | Ad | 5/29/11 | Al | LG | F |
| 170 | *Ziphius cavirostris* | M | Sad | 6/13/11 | D | TF | AA |
| 171 | *Delphinus delphis* | M | Ad | 7/4/11 | D | FT | F |
| 172 | *Globicephala macrorhynchus* | M | Jv | 7/23/11 | Al | TF | MA |
| 173 | *Tursiops truncatus* | F | C | 8/5/11 | D | TF | AA |
| 174 | *Globicephala macrorhynchus* | F | C | 8/24/11 | D | FT | MA |
| 175 | *Physeter macrocephalus* | F | Ad | 9/7/11 | D | TF | AA |
| 176 | *Globicephala macrorhynchus* | F | N | 9/10/11 | Al | FT | F |
| 177 | *Globicephala macrorhynchus* | F | Ad | 9/17/11 | D | GC | AA |
| 178 | *Stenella coeruleoalba* | M | Ad | 10/9/11 | D | GC | MA |
| 179 | *Stenella coeruleoalba* | M | Ad | 10/9/11 | D | FT | MA |
| 180 | *Ziphius cavirostris* | F | Ad | 11/1/11 | D | TF | AA |
| 181 | *Tursiops truncatus* | F | C | 11/5/11 | D | LG | MA |
| 182 | *Ziphius cavirostris* | M | Ad | 11/18/11 | D | GC | AA |
| 183 | *Globicephala macrorhynchus* | M | Sad | 12/2/11 | D | TF | F |
| 184 | *Tursiops truncatus* | M | Sad | 12/2/11 | D | LZ | F |
| 185 | *Stenella coeruleoalba* | F | Sad | 12/25/11 | D | GC | AA |
| 186 | *Kogia breviceps* | M | Ad | 1/3/12 | D | LG | AA |
| 187 | *Globicephala macrorhynchus* | F | C | 1/20/12 | D | TF | VAA |
| 188 | *Balaenoptera borealis* | F | Sad | 1/26/12 | D | GC | AA |
| 189 | *Stenella frontalis* | M | Sad | 2/5/12 | D | TF | F |
| 190 | *Physeter macrocephalus* | M | ND | 2/9/12 | D | EH | VAA |
| 191 | *Delphinus delphis* | F | Ad | 2/12/12 | D | GC | F |
| 192 | *Pseudorca crassidens* | M | Ad | 2/13/12 | D | LP | MA |
| 193 | *Stenella frontalis* | M | Ad | 2/21/12 | D | TF | F |
| 194 | *Stenella coeruleoalba* | F | Ad | 3/9/12 | D | LZ | F |
| 195 | *Delphinus delphis* | M | Sad | 3/12/12 | D | LZ | MA |
| 196 | *Delphinus delphis* | F | C | 3/12/12 | D | GC | AA |
| 197 | *Delphinus delphis* | F | C | 3/13/12 | D | FT | MA |
| 198 | *Kogia breviceps* | F | Ad | 3/26/12 | D | LZ | AA |
| 199 | *Stenella frontalis* | M | Ad | 4/5/12 | Al | FT | F |
| 200 | *Physeter macrocephalus* | M | C | 4/24/12 | D | FT | MA |
| 201 | *Delphinus delphis* | M | Ad | 4/27/12 | D | GC | MA |
| 202 | *Stenella coeruleoalba* | M | Sad | 4/28/12 | D | LZ | F |
| 203 | *Stenella coeruleoalba* | F | Ad | 5/2/12 | D | GC | F |
| 204 | *Delphinus delphis* | M | Ad | 5/10/12 | D | TF | MA |
| 205 | *Stenella frontalis* | M | Sad | 5/12/12 | D | TF | MA |
| 206 | *Globicephala macrorhynchus* | M | Jv | 5/19/12 | D | LGra | AA |
| 207 | *Ziphius cavirostris* | M | Ad | 5/20/12 | D | GC | VAA |
| 208 | *Stenella coeruleoalba* | F | Ad | 6/12/12 | D | FT | MA |
| 209 | *Physeter macrocephalus* | F | C | 6/21/12 | D | LZ | AA |
| 210 | *Globicephala macrorhynchus* | F | Ad | 6/27/12 | D | LZ | AA |
| 211 | *Ziphius cavirostris* | F | Ad | 7/13/12 | D | LGra | AA |
| 212 | *Megaptera novaeangliae* | ND | Jv | 7/20/12 | D | TF | VAA |
| 213 | *Kogia breviceps* | F | C | 7/26/12 | Al | TF | VF |
| 214 | *Globicephala macrorhynchus* | F | Sad | 9/23/12 | D | TF | F |
| 215 | *Physeter macrocephalus* | F | C | 10/19/12 | D | TF | AA |
| 216 | *Mesoplodon europaeus* | M | Ad | 10/21/12 | D | FT | AA |
| 217 | *Stenella coeruleoalba* | F | Sad | 10/22/12 | D | GC | MA |
| 218 | *Stenella frontalis* | M | Ad | 10/25/12 | D | FT | MA |
| 219 | *Grampus griseus* | M | Sad | 11/3/12 | D | FT | MA |
| 220 | *Tursiops truncatus* | M | Sad | 11/18/12 | D | GC | F |
| 221 | *Mesoplodon mirus* | M | Sad | 11/30/12 | D | EH | F |
| 222 | *Stenella frontalis* | M | Jv | 12/6/12 | Al | LZ | F |
| 223 | *Delphinus delphis* | F | Ad | 12/13/12 | D | GC | MA |
| 224 | *Tursiops truncatus* | M | Ad | 12/20/12 | D | LZ | VF |
