# Supplementary material for: Pathologic findings and causes of death of stranded cetaceans in the Canary Islands (2006-2012)
Source: PLoS One. 2018 Oct 5;13(10):e0204444. doi: 10.1371/journal.pone.0204444 (PMC6173391; doi:10.1371/journal.pone.0204444)
Supplement: S2 Table — CD: Cluster of differentiation; CK: cytokeratin; GFAP: glial fibrillary acid protein, NSE: neuron specific enolase, MAC387: myeloid/histiocytic antigen; CDV: canine distemper virus; HSV1: Herpes Simplex virus 1: HIER: Heat-induced epitope retrieval; ABC: Avidin-Biotin-Peroxidase complex. (DOCX) [file pone.0204444.s002.docx]

**S2 Table. Details of immunohistochemical analyses performed on formalin-fixed, paraffin-embedded tissue sections from selected cetacean species, including primary antibody (Ab), manufacturer, clonality, dilution, pretreatment, incubation, secondary Ab, manufacturer, and visualization system**. CD: Cluster of differentiation; CK: cytokeratin; GFAP: glial fibrillary acid protein, NSE: neuron specific enolase, MAC387: myeloid/histiocytic antigen; CDV: canine distemper virus; HSV1: Herpes Simplex virus 1: HIER: Heat-induced epitope retrieval; ABC: Avidin-Biotin-Peroxidase complex.

| **Primary Ab** | **Manufacturer** | **Clonality** | **Dilution** | **Pretreatment** | **Incubation** | **Secondary Ab** | **Manufacturer** | **Visualization** |
| --- | --- | --- | --- | --- | --- | --- | --- | --- |
| **CD3** | DAKO^c^ | Polyclonal | 1:100 | HIER pH6 | 50’ TA | Swine anti-rabbit | DAKO | EnVision |
| **CD79a** | DAKO | Monoclonal | 1:100 | HIER pH6 | 50’ TA | Rabbit anti-mouse | DAKO | EnVision |
| **AE1/AE3** | DAKO | Monoclonal | 1:100 | HIER pH6 | 50’ TA | Rabbit anti-mouse | DAKO | EnVision |
| **CK7** | DAKO | Monoclonal | 1:20 | Pronase 10’ | 50’ TA | Rabbit anti-mouse | DAKO | EnVision |
| **CK14** | NOVOCASTRA^d^ | Monoclonal | 1:20 | HIER pH6 | 50’ TA | Rabbit anti-mouse | DAKO | EnVision |
| **CK20** | DAKO | Monoclonal | 1:20 | HIER pH9 | 50’ TA | Rabbit anti-mouse | DAKO | EnVision |
| **Calponin** | DAKO | Monoclonal | 1:400 | HIER pH6 | 18 h, 4ºC | Rabbit anti-mouse | DAKO | ABC |
| **Calretinin** | DAKO | Monoclonal | 1:100 | HIER pH9 | 18 h, 4ºC | Rabbit anti-mouse | DAKO | ABC |
| **S-100** | DAKO | Polyclonal | 1:100 | Pronase 10’ | 30’ TA | Swine anti-rabbit | DAKO | EnVision |
| **GFAP** | NEOMARKERS^e^ | Monoclonal | 1:100 | Pronase 10’ | 50’ TA | Rabbit anti-mouse | DAKO | EnVision |
| **Neurofilament** | NEOMARKERS | Monoclonal | 1:1000 | HIER pH6 | 50’ TA | Rabbit anti-mouse | DAKO | EnVision |
| **NSE** | NEOMARKERS | Monoclonal | 1:100 | HIER pH6 | 18 h, 4ºC | Rabbit anti-mouse | DAKO | EnVision |
| **Lysozyme** | DAKO | Polyclonal | 1:400 | Pronase 10’ | 18 h, 4ºC | Swine anti-rabbit | DAKO | ABC |
| **MAC387** | DAKO | Monoclonal | 1:100 | Pronase 10’ | 18 h, 4ºC | Rabbit anti-mouse | DAKO | ABC |
| ***Erysipelothrix rhusiopathiae*** | *In house* (from animal no. 133) | Polyclonal | 1:100 | Pronase 10’ | 18 h, 4ºC | Rabbit anti-mouse | DAKO | ABC |
| ***Morbillivirus* ^a^** | VMRD^f^ | Monoclonal | 1:100 | HIER^h^ | 18 h, 4ºC | Rabbit anti-mouse | DAKO | ABC |
| ***Herpesvirus* ^b^** | Abcam^g^ | Polyclonal | 1:200 | HIER^h^ | 18 h, 4ºC | Swine anti-mouse | DAKO | ABC |
| ***Toxoplasma gondii*** | VMRD | Polyclonal | 1:400 | HIER^i^ | 18 h, 4ºC | Rabbit anti-goat | DAKO | ABC |

^a^ Cetacean morbillivirus (CeMV). Commercially available monoclonal Ab for CDV nucleoprotein.

^b^ HSV1

^c^ DAKO, Glostrup, Denmark

^d^ NOVOCASTRA

^e^ NEOMARKERS

^f^ VMRD

^g^ Abcam

^h^ Autoclave

^i^ Citrate
