# Supplementary material for: Pathologic findings and causes of death of stranded cetaceans in the Canary Islands (2006-2012)
Source: PLoS One. 2018 Oct 5;13(10):e0204444. doi: 10.1371/journal.pone.0204444 (PMC6173391; doi:10.1371/journal.pone.0204444)
Supplement: S3 Table — (DOCX) [file pone.0204444.s003.docx]

| **S3 Table. Tissues submitted for microbiological analysis and results from a subset of 224 stranded and necropsied cetaceans.** | | |
| --- | --- | --- |
| **Case No** | **Organ cultured** | **Results** |
| 36 | Lung | *Streptococcus phocae* |
|  | Brain |  |
|  | Adrenal |  |
| 63 | Cerebrum | *Aspergillus fumigatus* |
| 64 | Lung | No growth |
|  | Brain |  |
|  | Kidney |  |
|  | Liver | *Photobacterium damselae* |
| 65 | Liver | No growth |
|  | Lung |  |
|  | Brain | *Enterococcus faecalis* |
| 67 | Placenta | *Chryseomonas luteola* |
|  | Liver | No growth |
|  | Kidney |  |
|  | Lung |  |
|  | Brain |  |
|  | Uterus |  |
|  | Umbilical cord | Polymicrobial (not significant) |
| 68 | Liver | *Pseudomonas taetrolens* |
|  | Kidney | *Aeromonas hydrophila/caviae* |
|  | Lung | *Pseudomonas putida, Aeromonas hydrophila/caviae* |
|  | Brain | *Aeromonas salmonicida, Pseudomonas putida, Pseudomonas taetrolens* |
| 70 | Lung | No growth |
| 75 | Liver | No growth |
|  | Kidney |  |
|  | Lung |  |
|  | Brain |  |
| 76 | Liver | No growth |
|  | Brain | *Fusobacterium varium* |
|  | Intestine | *Clostridium sordellii, Clostridium bifermentans* |
| 90 | Brain | *Photobacterium damselae* |
| 95 | Heart | No growth |
|  | Brain |  |
| 100 | Heart | *Enterococcus faecalis* |
| 101 | Spleen | No growth |
|  | Lung | *Vagococcus fluviales* |
|  | Brain | *Moraxella* sp. |
| 109 | Lung | *Enterococcus faecalis* |
|  | Brain |  |
|  | Liver |  |
| 110 | Lung | No growth |
|  | Brain |  |
| 114 | Lung | *Clostridium perfringens* |
|  | Brain | *Photobacterium damselae, Clostridium perfringens* |
| 115 | Liver | No growth |
|  | Lung |  |
|  | Mesenteric lymph node |  |
| 117 | Lung | No growth |
|  | Brain |  |
|  | Mediastinal lymph node |  |
| 118 | Lung | No growth |
|  | Brain | *Fusobacterium varium* |
| 125 | Lung | *Alcaligenes faecalis* sp. *faecalis* |
|  | Brain | *Fusobacterium varium* |
|  | Skin | No growth |
| 127 | Liver | No growth |
|  | Lung |  |
|  | Brain |  |
|  | Kidney | *Pseudomonas aeruginosa* |
| 131 | Liver | No growth |
|  | Kidney | *Enterococcus faecalis* |
|  | Lung | *Microbacterium resistens* |
| 133 | Liver | *Erysipelothrix rhusiopathiae* |
|  | Lung |  |
|  | Mesenteric lymph node |  |
| 138 | Mesenteric lymph node | *Staphylococcus epidermidis* |
|  | Liver | No growth |
|  | Spleen |  |
|  | Kidney |  |
| 147 | Kidney | *Photobacterium damselae, Aerococcus viridans* |
|  | Lung | *Photobacterium damselae* |
|  | Brain |  |
|  | Spleen | No growth |
| 149 | Brain | *Staphylococcus pseudointermedius* |
|  | Kidney | *Staphylococcus intermedius* |
|  | Cardiac blood | No growth |
|  | Liver |  |
|  | Spleen |  |
|  | Lung |  |
|  | Mesenteric lymph node |  |
| 151 | Spleen | No growth |
|  | Brain |  |
|  | Mesenteric lymph node |  |
|  | Kidney | *Clostridium sordelli*, *Cl*. *perfringens* |
|  | Lung | *Acinetobacter johnsonii* |
|  | Uterus | *Photobacterium damselae* |
| 152 | Mammary gland, vagina | *Mycoplasma* sp.^a^ |
| 165 | Mammary gland | *Mycoplasma* sp.^a^ |
|  | Vulva |  |
|  | Auditory canal |  |
|  | Eye |  |
|  | Lung |  |
|  | Atlanto-occipital arthritis |  |
|  | Pleura |  |
| 168 | Lung | *Kocuria rosae* |
|  | Brain | *Macrococcus caseolyticus* |
|  | Mediastinal lymph node | *Staphylococcus warnei* |
|  | Mesenteric lymph node | *Macrococcus caseolyticus* |
|  | Spleen | *Staphylococcus kloosii* |
|  | Kidney | No growth |
|  | Skin |  |
|  | Cardiac blood |  |
|  | Liver |  |
|  | Stomach |  |
|  | Urine |  |
| 171 | Spleen | No growth |
|  | Kidney |  |
|  | Lung |  |
|  | Brain |  |
|  | Intestine |  |
|  | Mesenteric lymph node |  |
|  | Prescapular lymph node |  |
| 177 | Lung | *Brevibacterium* spp. |
|  | Brain | *Brevibacterium* spp. |
|  | Reproductive system | *Rhodococcus* spp; *Clostridium perfringens* |
|  | Mesenteric lymph node | *Sphingomonas paucimobilis, Clostridium perfringens* |
|  | Mediastinal lymph node | *Clostridium perfringens, Brevibacterium* sp. |
|  | Liver | No growth |
|  | Kidney |  |
| 178 | Pleura | Mollicutes*^a^ |
| 181 | Liver | No growth |
|  | Kidney |  |
|  | Lung |  |
|  | Brain |  |
|  | Intestine |  |
|  | Mediastinal lymph node |  |
|  | Mammary gland |  |
| 183 | Mammary gland | Mollicutes*^a^ |
| 184 | Lung | *Stenotrophomonas maltophilia, Corynebacterium bovis* |
|  | Larynx | No growth |
|  | Brain |  |
|  | Blowhole | Mollicutes*^a^ |
| 186 | Blowhole | *Mycoplasma* sp.^a^ |
| 188 | Mouth | Mollicutes*^a^ |
| 189 | Liver | *Erysipelothrix rhusiopathiae* |
|  | Lung |  |
|  | Mesenteric lymph node |  |
|  | Brain |  |
|  | Kidney |  |
| 191 | Mesenteric lymph node | *Clostridium perfringens* |
|  | Urine | *Clostridium sordelli* |
|  | Abdominal coagule | *Escherichia coli* |
|  | Liver | No growth |
|  | Spleen |  |
|  | Kidney |  |
|  | Lung |  |
|  | Brain |  |
|  | Uterus |  |
|  | Mediastinal lymph node |  |
|  | Mammary gland |  |
| 193 | Kidney | *Arthrobacter luteus* |
|  | Liver | No growth |
|  | Lung |  |
|  | Spleen |  |
|  | Brain |  |
|  | Mesenteric lymph node |  |
|  | Retroperitoneal lymph node |  |
|  | Gingiva |  |
| 199 | Liver | *Stenotrophomonas maltophilia* |
|  | Mesenteric lymph node | *Microbacterium resistens* |
|  | Mediastinal lymph node | *Brevundimonas diminuta* |
|  | Spleen | *Escherichia coli* |
|  | Kidney | *Gemella haemolysans, Enterococcus hirae* |
|  | Lung | No growth |
|  | Brain |  |
| 201 | *Cardiac blood* | *Wohlfartiimonas chitiniclastica* |
|  | Vegetative endocarditis |  |
| 211 | Lung | Mollicutes*^a^ |
| 214 | Cardiac blood | No growth |
|  | Lung |  |
|  | Brain |  |
|  | Anus | Mollicutes*^a^ |
| 221 | Eye  Lung | *Acholeplasma axanthum*^a^ |
| 222 | Anus | Mollicutes*^a^ |
| 224 | Brain | *Aerococcus viridans,* *Clostridium* spp. |

* No further identification.

^a^ Mollicutes’ culture used RPMI-1640 media. These results are part of a parallel and collaborative PhD project [*Vega Orellana, Orestes. 2014. Estudio de microorganismos de la clase Mollicutes en organismos marinos. PhD Thesis. Institute of Animal Health and Food Security, University of Las Palmas of Gran Canaria, Spain*]. <https://acceda.ulpgc.es:8443/bitstream/10553/12198/4/0701361_00000_0000.pdf>
