# Supplementary material for: Pathologic findings and causes of death of stranded cetaceans in the Canary Islands (2006-2012)
Source: PLoS One. 2018 Oct 5;13(10):e0204444. doi: 10.1371/journal.pone.0204444 (PMC6173391; doi:10.1371/journal.pone.0204444)
Supplement: S4 Table — (DOCX) [file pone.0204444.s004.docx]

**S4 Table**. **Main morphologic and etiologic diagnoses in animals included in ‘pathology associated with good nutritional status’**.

| **No** | **Morphologic diagnoses** | **Etiologic diagnoses** |
| --- | --- | --- |
| 1 | Suppurative bronchopneumonia; lymphoplasmacytic periportal hepatitis. | Infectious bronchopneumonia and hepatitis |
| 2 | Lymphoplasmacytic bronchointerstitial pneumonia; uterine leiomyoma and fibroleiomyoma; urinary bladder fibroma. | Senile changes; Multiple neoplasia |
| 5 | Fibrinonecrotizing vaginitis and endometritis. | Infectious vaginitis and endometritis |
| 7 | Lymphocytic poliomyelomeningitis and encephalitis. | Infectious poliomyelomeningitis and encephalitis |
| 8 | Suppurative bronchopneumonia with interstitial and bronchial sclerosis; lymphoplasmacytic pterygoid sinusitis with intralesional *Nasitrema* sp. and *Stenurus* sp. | Infectious bronchopneumonia; parasitic sinusitis; systemic parasitosis |
| 17 | Lymphoplasmacytic meningoencephalitis; multicentric lymphoid depletion. | Infectious meningoencephalitis |
| 18 | Lymphoplasmacytic bronchointerstitial and exudative pneumonia with bronchial sclerosis; lymphoplasmacytic periportal hepatitis; lymphoplasmacytic interstitial nephritis; lymphoplasmacytic endometritis; pulmonary fat embolism. | Infectious bronchointerstitial pneumonia, hepatitis, nephritis and endometritis |
| 26 | Lymphoplasmacytic meningoencephalitis; pterygoid sinusitis with intralesional *Nasitrema* sp.; lymphoplasmacytic myocarditis. | Infectious meningitis and myocarditis; Systemic parasitosis |
| 36 | Lymphoplasmacytic and neutrophilic meningochoroiditis with bacterial emboli; acute interstitial pneumonia with bacterial emboli; vegetative endocarditis; lymphoplasmacytic and necrotizing cortical adrenalitis; pyogranulomatous dermatitis with intralesional and intravascular gram-positive bacteria. | *Streptococcus phocae* septicemia and morbilliviral meningochoroiditis |
| 41 | Suppurative bronchopneumonia with fibrin; chronic glomerulopathy. | Infectious bronchopneumonia |
| 44 | Lymphoplasmacytic and histiocytic meningomyelitis, meningoencephalitis, perineuritis and choroiditis. | Infectious meningoencephalomyelitis |
| 46 | Lymphohistiocytic myocarditis and encephalomyelitis with intralesional protozoal cysts (*Toxoplasma* *gondii*). | Systemic toxoplasmosis |
| 50 | Thalamic high-grade astrocytoma (glioblastoma multiforme) | Primary cerebral neoplasia |
| 56 | Multifocal epi- and subdural hemorrhages; spongy leukomyeloencephalopathy with axonal degeneration, perivascular edema and astrocytosis. | Spongy leukomyeloencephalopathy of unknown origin. |
| 63 | Pyogranulomatous and necrotizing meningoencephalitis with vasculitis, thrombosis and intralesional hyphae. | Fungal meningoencephalitis |
| 65 | Intestinal torsion with venous mesenteric infarction and enteric necrosis; fibrinous peritonitis. | Intestinal torsion; septicemia |
| 67 | Lymphoplasmacytic meningoencephalitis; severe pulmonary edema; subendocardial and myocardial hemorrhage. | Infectious meningoencephalitis |
| 69 | Hemoabdomen | Idiopathic hemorrhage |
| 76 | Lymphoplasmacytic meningitis; pterygoid sinusitis with intralesional *Nasitrema* sp. and *Crassicauda* sp.; lymphoplasmacytic interstitial nephritis with rare pyogranulomas; granulomatous bronchitis with bronchial sclerosis and intralesional nematodes. | Infectious meningitis; Systemic parasitosis |
| 81 | Lymphoplasmacytic meningitis; lymphoplasmacytic and exudative bronchointerstitial pneumonia; granulomatous and eosinophilic lymphadenitis; purulent pterygoid sinusitis with intralesional *Crassicauda* sp. and *Nasitrema* sp. | Infectious meningitis and bronchointerstitial pneumonia; Systemic parasitosis |
| 83 | Pyogranulomatous and obstructive urethritis with intralesional  *Crassicauda* sp.; hemoabdomen; hemopericardium. | Parasitic obstructive urethritis |
| 85 | Pyogranulomatous pneumonia with sclerosing bronchitis and intralesional nematodes; pyogranulomatous fascitis with intralesional *Crassicauda* sp. | Systemic parasitosis |
| 90 | Transmural fibrinonecrotizing and suppurative enteritis and peritonitis. | Enterotoxemia; Septicemia |
| 92 | Transmural dissecting neutrophilic and eosinophilic endarteritis with necrosis, fibrosis, intraluminal nematodes, and thrombosis; severe obstructive ureteritis and nephritis with intralesional *Crassicauda* sp. | Arterial and renal crassicaudiasis |
| 95 | Fibrinosuppurative pericarditis, myocarditis and aortic periarteritis; lymphoplasmacytic and histiocytic meningitis and choroiditis. | Infectious pericarditis, myocarditis, periarteritis and meningitis (septicemia) |
| 100 | Fibrinous aortic (thoracic, abdominal) arteritis and pulmonary arterial necrosis; severe proliferative cholangitis with intralesional *Brachycladiidae*; eosinophilic and catarrhal bronchitis with intralesional nematodes. | Infectious aortic arteritis |
| 106 | Lymphoplasmacytic bronchointerstitial pneumonia with intralesional *Nasitrema* sp. ova; pyogranulomatous gastritis with intralesional *Pholeter gastrophilus*; granulomatous pancreatitis and ductitis with intralesional *Brachycladiidae*; Granulomatous panniculitis. | Systemic parasitosis |
| 109 | Intestinal perforation and septic peritonitis | Septic peritonitis |
| 114 | Lymphoplasmacytic and histiocytic meningitis; bronchointerstitial and exudative pneumonia with scattered granulomas and intralesional nematodes; hemopericardium; hemothorax. | Infectious meningitis and bronchointerstitial pneumonia; Systemic parasitosis |
| 115 | Fibrinosuppurative and necrotizing pleuropneumonia; cholangiocarcinoma; hemoabdomen; uterine hemorrhage. | Infectious pleuropneumonia; Hepatic neoplasia |
| 117 | Lymphoplasmacytic meningomyeloencephalitis, perineuritis and choroiditis with intralesional Gram-negative bacteria; lymphoplasmacytic bronchointerstitial pneumonia with scattered pyogranulomas, necrosis, and type II pneumocyte hyperplasia. | Infectious myelomeningoencephalitis, perineuritis, choroiditis and bronchointerstitial pneumonia |
| 118 | Fibrosing mesenteric arteritis with aneurysms and thrombosis. | Arterial crassicaudiasis |
| 126 | Lymphohistiocytic and neutrophilic meningitis with necrosis; lymphoplasmacytic myocarditis; lymphoplasmacytic adrenalitis. | Systemic toxoplasmosis; Systemic parasitosis |
| 131 | Lymphohistiocytic and necrotizing meningoencephalitis, gastrointestinal leiomyositis, and adrenalitis with intralesional protozoal cysts (*Toxoplasma* *gondii*). | Systemic toxoplasmosis |
| 133 | Systemic bacteremia; acute neutrophilic embolic glomerulitis and nephritis with hemorrhage; lymphoplasmacytic and neutrophilic myocarditis; lymphoplasmacytic bronchointerstitial pneumonia. | *Erysipelothrix rhusiopathiae* septicemia |
| 144 | Lymphoplasmacytic choroiditis; maxillary fracture; multisystemic hemorrhage; lymphoplasmacytic cholangiohepatitis with sinusoidal plasmacytosis and lymphocytolysis; pyogranulomatous and eosinophilic lymphadenitis with sinus erythrocytosis and intralesional ciliate protozoa. | Infectious choroiditis, and hepatitis; ciliate protozoal lymphadenitis; Systemic parasitosis; Trauma |
| 146 | Necrotizing bronchopneumonia with alveolar keratin spicules; multifocal hepatic venous thrombosis with necrosis (infarcts) | Infectious bronchopneumonia; Disseminated intravascular coagulation |
| 147 | Systemic gas embolism; focal pleural rupture and pneumothorax; lymphoplasmacytic and histiocytic choroiditis; ulcerative glossitis with intralesional bacteria and myocyte necrosis; fibrinonecrotizing and ulcerative pharyngitis with vasculitis, hemorrhage, thrombosis, and intralesional bacteria; multicentric benign epithelial inclusions (mesothelial origin) | Gas embolism; Infectious pharyngitis, choroiditis and glossitis |
| 149 | Lymphoplasmacytic meningoencephalitis; suppurative bronchopneumonia; fibrinosuppurative and ulcerative vulvitis; mesangiocapillary glomerulopathy; lymphoplasmacytic interstitial nephritis; ulcerative stomatitis and glossitis. | Infectious meningoencephalitis, bronchopneumonia and vulvitis |
| 151 | Suppurative and necrotizing bronchopneumonia with intralesional nematodes; pterygoid sinusitis with intralesional *Nasitrema* sp.; granulomatous lymphadenitis with intralesional *Brachycladiidae* ova. | Infectious bronchopneumonia; Systemic parasitosis |
| 152 | Lymphoplasmacytic meningoencephalomyelitis and poliomyelitis; fibrinosuppurative and necrotizing bronchopneumonia with sclerosing bronchiolitis and chondrolysis; pyogranulomatous and eosinophilic lymphadenitis with necrosis and occasional syncytia; lymphoplasmacytic interstitial nephritis with fibrosis, nephron atrophy and glomerulosclerosis. | Infectious meningoencephalitis, poliomyelitis, bronchopneumonia, and nephritis; Multicentric lymphadenitis |
| 157 | Granulomatous and necrotizing meningoencephalitis with intralesional *Nasitrema* sp. ova; bronchointerstitial pneumonia with intralesional nematodes, *Nasitrema* sp. ova, bacteria, and marked edema; multisystemic thrombosis. | *Nasitrema* sp. meningoencephalitis |
| 159 | Lymphocytic encephalitis; necrosuppurative vaginitis with intralesional bacteria; lymphoplasmacytic bronchointerstitial pneumonia with hemorrhage and alveolar bacteria; lymphoplasmacytic interstitial nephritis with intravascular bacteria and hemorrhage; multifocal adrenocortical hemorrhage with intravascular bacteria; lymphocytic choroiditis; multifocal myocardial hemorrhage with acute myodegeneration and necrosis, and edema. | Bacteremia (septicemia) |
| 161 | Suppurative bronchopneumonia with type II pneumocyte hyperplasia and edema. | Infectious bronchopneumonia |
| 163 | Gas encephalopathy with hemorrhage and perivascular edema; pigmentary tubulonephrosis; pterygoid sinusitis with intralesional *Crassicauda* sp. and *Nasitrema* sp. | Gas embolism; Systemic parasitosis |
| 167 | Multifocal necrotizing hepatitis with intralesional bacteria; chronic pleuritis with fibrosis and parietal and diaphragmatic pleural adhesions; chronic fibrosing mesenteric arteritis with aneurysms, thrombosis and hemorrhage; severe obstructive ureteritis and nephritis with intralesional *Crassicauda* sp. | Infectious necrotizing hepatitis, chronic pleuritis; Arterial and renal crassicaudosis |
| 171 | Lymphoplasmacytic meningoencephalitis and poliomyelitis; Multifocal lymphoplasmacytic myocarditis; multifocal myocardial fibrosis and cardiomyocyte loss (infarcts); severe lymphoplasmacytic tubulointerstitial nephritis; lymphoplasmacytic adrenalitis; lymphoplasmacytic pterygoid sinusitis with intralesional nematodes. | Infectious meningoencephalitis; Systemic parasitosis |
| 172 | Lymphoplasmacytic meningoencephalitis and poliomyelitis; pterygoid sinusitis with intralesional *Stenurus* sp. | Infectious meningoencephalitis and poliomyelitis; *Stenurus* sp pterygoid sinusitis |
| 177 | Granulomatous gastritis with intralesional *P. gastrophilus*; multifocal pulmonary granulomas; multifocal urinary bladder hemorrhage; uterine leiomyoma. | Systemic parasitosis; Uterine neoplasia |
| 178 | Suppurative bronchopneumonia with intralesional nematodes; granulomatous gastritis with intralesional *P. gastrophilus*; pyogranulomatous lymphadenitis with intralesional nematodes. | Systemic parasitosis |
| 180 | Fibrosing thoracic and abdominal aortic arteritis with aneurysms, thrombosis and hemorrhage; severe obliterative urethritis, nephritis, and renal arteritis with intralesional *Crassicauda* sp. | Arterial and renal crassicaudiasis |
| 181 | Lymphocytic choroiditis; necroticosuppurative bronchopneumonia with intralesional nematodes; hydropericardium; hydrothorax; ascites. | Infectious bronchopneumonia and choroiditis |
| 188 | Hemorrhagic and obstructive enteritis with numerous intralesional *Bolbosoma* sp.; interstitial pneumonia with thrombosis. | *Bolbosoma* sp. hemorrhagic enteritis |
| 189 | Systemic bacteremia; acute neutrophilic embolic glomerulitis and nephritis; acute lymphoplasmacytic interstitial pneumonia. | *Erysipelothrix rhusiopathiae* septicemia |
| 191 | Uterine rupture; hemoperitoneum | Uterine rupture (hypovolemic shock) |
| 192 | Pyogranulomatous enteritis with intralesional *Bolbosoma* sp.; suppurative pterygoid sinusitis with intralesional *Stenurus* sp. | Systemic parasitosis |
| 193 | Suppurative bronchopneumonia with intrabonchiolar nematodes; lymphoplasmacytic cholangiohepatitis with necrosis; interstitial lymphoplasmacytic nephritis; ulcerative glossitis, stomatitis and gingivitis; generalized reactive lymphadenomegaly; multinodal pyogranulomatous lymphadenitis; lymphoplasmacytic adrenalitis. | Infectious bronchopneumonia, hepatitis and nephritis; Systemic parasitosis |
| 195 | Multiple mandibular fractures with hemorrhage; suppurative pterygoid sinusitis with intralesional *Nasitrema* sp. and nematodes with migration through vestibulocochlear nerves; multifocal meningeal cerebral and cerebellar edema and hemorrhage; ascites; acute skeletal and myocardial rhabdomyolysis. | Systemic parasitosis; Trauma; Live-stranding stress syndrome |
| 198 | Granulomatous and eosinophilic thoracic and cervical dermatitis, panniculitis, fasciitis and myositis, and vascular obliteration (*rete mirabili*) with intralesional *Crassicauda* sp.; acute alveolar edema and hemorrhage; medullary hemorrhage; ascites; hydropericardium. | Systemic parasitosis |
| 202 | Lymphoplasmacytic meningoencephalitis and poliomyelitis with intranuclear and intracytoplasmic eosinophilic inclusion bodies, acute necrosis and hemorrhage; interstitial pneumonia with syncytia and type II pneumocyte hyperplasia; multicentric lymphoid depletion and lymphocytolysis. | Acute systemic morbilivirosis; Systemic parasitosis |
| 204 | Pyogranulomatous pterygoid sinusitis with intralesional *Nasitrema* sp.; suppurative bronchopneumonia with intralesional nematodes; systemic bacterial emboli; esophageal fistula. | Bacteremia (septicemia); Systemic parasitosis |
| 205 | Lymphoplasmacytic meningoencephalitis, meningomyelitis and polirradiculoneuritis with fibrinoid vascular necrosis and perivascular cuffing; focally extensive hemorrhage in *rete mirabilis*. | Infectious meningoencephalitis, meningomielitis and polirradiculoneuritis; Systemic parasitosis |
| 207 | Severe chronic fibrosing arteritis with aneurysms, thrombosis, fibrin, hemorrhage; severe obliterative urethritis, nephritis and renal arteritis with intralesional *Crassicauda* sp. | Arterial and renal crassicaudiasis |
| 208 | Lymphoplasmacytic leptomeningoencephalitis and myelitis; transmural pyogranulomatous gastritis with focal perforation and septic fibrinosuppurative peritonitis; hepatic abscess; systemic trematode ova and bacterial emboli. | Septic peritonitis (septicemia); Systemic trematodiasis |
| 214 | Lymphoplasmacytic meningitis; suppurative pterygoid sinusitis with intralesional *Stenurus* sp.; skeletal acute rhabdomyolysis; suppurative bronchopneumonia with alveolar bacteria and type II pneumocyte hyperplasia. | Infectious leptomeningitis and bronchopneumonia |
| 217 | Fibrinosuppurative and necrotizing pleuropneumonia; Pyothorax; fibrinosuppurative and necrotizing pulmonary and aortic serositis; Focal chronic hepatic thrombosis with recanalization and chronic infarct. | Infectious pleuropneumonia; Septicemia; Systemic thrombosis |
| 220 | Pyogranulomatous and necrotizing meningoencephalitis and neuritis with intralesional *Nasitrema* sp. ova; lymphoplasmacytic and ulcerative pterygoid sinusitis with intralesional *Nasitrema* sp. | *Nasitrema* sp meningoencephalitis; Systemic parasitosis |
| 222 | Lymphoplasmacytic meningoencephalitis; lymphoplasmacytic bronchointerstitial pneumonia; multicentric lymphoid depletion. | Infectious meningoencephalitis; Systemic parasitosis |
| 223 | Lymphoplasmacytic meningoencephalitis; pigmentary tubulonephrosis; multicentric lymphoid depletion; lymphoplasmacytic adrenalitis with intranuclear eosinophilic inclusion bodies (compatible with *Herpesvirus*). | Infectious meningoencephalitis and adrenalitis |
| 224 | Granulomatous and necrotizing meningoencephalitis and neuritis with intralesional *Nasitrema* sp.; granulomatous pterygoid sinusitis and otitis; pigmentary tubulonephrosis; acute skeletal and myocardial rhabdomyolysis. | *Nasitrema* sp. pterygoid sinusitis, otitis media and interna and meningoencephalitis; Live-stranding stress syndrome |
