# Supplementary material for: Pathologic findings and causes of death of stranded cetaceans in the Canary Islands (2006-2012)
Source: PLoS One. 2018 Oct 5;13(10):e0204444. doi: 10.1371/journal.pone.0204444 (PMC6173391; doi:10.1371/journal.pone.0204444)
Supplement: S5 Table — (DOCX) [file pone.0204444.s005.docx]

**S5 Table**. **Main morphologic and etiologic diagnoses in animals included in ‘natural pathology associated with significant loss of nutritional status’**.

| **No** | **Morphologic diagnosis** | **Etiologic diagnosis** |
| --- | --- | --- |
| 4 | Lymphoplasmacytic meningoencephalitis, myelitis and polirradiculitis | Infectious meningoencephalitis, myelitis and polirradiculitis |
| 6 | Peritonitis and hemoabdomen due to ruptured hepatic abscess; Hepatocellular carcinoma | Septic peritonitis (septicemia); Hepatic neoplasia |
| 16 | Mild glomerulopathy with tubular proteinosis and hyaline casts; testicular atrophy; Marked subcutaneous (anogenital) parasitization by *Phyllobothrium delphini* merocercoids; Marked peritoneal parasitization *Monorygma grimaldi* merocercoids | Systemic parasitosis; Senile changes |
| 22 | Pulmonary interstitial and pleural fibrosis with alveolar edema and hemorrhage; hepatic lipidosis; chronic lymphoplasmacytic cholangitis; focal subcutaneous and fascial abscess with intralesional *Crassicauda* sp.; lymphoplasmacytic and histiocytic enteritis. | Systemic parasitosis. |
| 28 | Lymphoplasmacytic meningoencephalitis, myelitis, polirradiculoneuritis and neurohypophisitis | Infectious meningoencephalitis and neurohypophisitis |
| 33 | Lymphoplasmacytic encephalitis; Lymphoplasmacytic bronchointerstitial pneumonia with multifocal pyogranulomas; lymphoplasmacytic cortical adrenalitis | Infectious encephalitis, bronchointerstitial pneumonia and adrenalitis; Systemic parasitosis. |
| 39 | Bronchointerstitial and exudative pneumonia with fibrin and intralesional bacteria; fibrinonecrotizing diaphragmatic myositis and fascitis with thrombosis and intralesional bacteria; lymphoplasmacytic choroiditis. | Infectious choroiditis and bronchointerstitial pneumonia; Systemic parasitosis. |
| 43 | Lymphoplasmacytic and histiocytic meningoencephalomyelitis; lymphoplasmacytic and histiocytic bronchointerstitial pneumonia with fibrosis, bronchial sclerosis, scattered granulomas with intralesional nematodes and bacteria; lymphoplasmacytic adrenalitis. | Infectious meningoencephalitis, bronchopneumonia and adrenalitis |
| 45 | Pyogranulomatous and necrotizing meningoencephalitis with intralesional *Nasitrema* sp.; Purulent sinusitis with intralesional *Nasitrema* sp. | *Nasitrema* sp. pterygoid sinusitis and meningoencephalitis |
| 49 | Primary uterine T-cell lymphoma with metastasis | Primary uterine neoplasia with metastasis |
| 53 | Lymphoplasmacytic bronchointerstitial pneumonia with scattered pyogranulomas and intralesional nematodes; Neutrophilic and lymphoplasmacytic cholangiohepatitis; Mesangiocapillary glomerulopathy; Pyogranulomatous and lymphoplasmacytic orchitis with intralesional bacteria and pampiniform plexus thrombosis. | Infectious bronchopneumonia, cholangiohepatitis; Systemic parasitosis. |
| 54 | Keratinized stomach perforation and peritonitis; Multisystemic thrombosis. | Septic peritonitis |
| 59 | Suppurative mastitis | Infectious mastitis; Systemic parasitosis. |
| 61 | Lymphoplasmacytic encephalitis | Infectious encephalitis; Systemic parasitosis. |
| 68 | Glossitis, palatitis, esophagitis and gastritis, and dermatitis with rare intranuclear epithelial eosinophilic inclusion bodies (compatible with herpesvirus); intravascular bacteria, leukocytosis and intravascular coagulation. | Viral systemic infection and bacterial septicemia |
| 70 | Lymphoplasmacytic and neutrophilic periportal hepatitis with scattered hepatocellular necrosis; lymphoplasmacytic and granulomatous bronchointerstitial pneumonia with intralesional nematodes; nephrolithiasis; necrotizing pulmonary lymphadenitis and lymphoid depletion. | Infectious hepatitis; Parasitic bronchointerstitial pneumonia and lymphadenitis |
| 73 | Granulomatous fascitis with intralesional *Crassicauda* sp.; suppurative mastitis and galactophoritis with intralesional *Crassicauda* sp. | Systemic parasitosis |
| 75 | Lymphoplasmacytic leptomeningitis and neuritis. | Infectious meningitis |
| 77 | Lymphoplasmacytic meningitis and spinal perineuritis; bronchointerstitial pneumonia with epithelial necrosis; lymphoplasmacytic adrenalitis; ulcerative esophagitis and pharyngitis. | Infectious meningitis; Systemic viral infection |
| 79 | Lymphoplasmacytic bronchointerstitial pneumonia with intralesional nematode eggs; granulomatous interstitial nephritis; granulomatous mesenteric lymphadenitis; pyogranulomatous mastitis with intralesional *Crassicauda* sp.; Subcutaneous temporomandibular and prescapular hemorrhage. | Systemic parasitosis |
| 80 | Lymphoplasmacytic periportal hepatitis with scattered hepatocellular necrosis; ulcerative dermatitis, panniculitis, fasciitis and myositis with intralesional *Crassicauda* sp.;  focal pyogranulomatous lymphadenitis with intralesional trematodes; pyogranulomatous lymphadenitis with intralesional *Crassicauda* sp.; severe axial muscle atrophy. | Systemic parasitosis |
| 84 | Suppurative encephalitis; suppurative pterygoid sinusitis with intralesional *Crassicauda* sp.; lymphoplasmacytic adrenalitis. | Infectious encephalitis and adrenalitis |
| 91 | Lymphoplasmacytic meningoencephalitis; suppurative pterygoid sinusitis with intralesional *Nasitrema* sp.; fibrinosuppurative bronchopneumonia with intralesional nematodes. | Infectious meningoencephalitis, bronchopneumonia; Systemic parasitosis. |
| 94 | Neutrophilic and histiocytic encephalitis with necrosis; Pyogranulomatous lymphadenitis with intralesional ciliate protozoa; pyogranulomatous and ulcerative dermatitis with intralesional ciliate protozoa; suppurative and ulcerative pharyngitis and gingivitis. | Infectious encephalitis; Systemic ciliate protozoosis |
| 96 | Lymphohistiocytic meningoencephalitis, myocarditis, gastric and intestinal leiomyositis and adrenalitis with intralesional protozoal cysts (*Toxoplasma* *gondii*). | Systemic toxoplasmosis |
| 97 | Lymphoplasmacytic encephalitis. | Infectious encephalitis |
| 101 | Fibrinosuppurative bronchopneumonia with intralesional bacteria; multifocal random lymphoplasmacytic hepatitis. | Infectious bronchopneumonia and hepatitis |
| 108 | Erosive-ulcerative stomatitis, glossitis and pharyngitis; Multisystemic hemorrhage; Focal hepatic subcapsular hematoma; lymphoplasmacytic bronchointerstitial pneumonia; suppurative and histiocytic myositis and edema. | Infectious stomatitis, glossitis and pharyngitis; Cutaneous poxviral infection; Systemic parasitosis |
| 110 | Fibrinosuppurative and necrotizing pleuropneumonia (right lung); pyothorax; fibrinosuppurative peritonitis; fibrinosuppurative pericarditis. | Infectious pleuropneumonia, pyothorax, peritonitis and pericarditis |
| 125 | Severe suppurative pterygoid sinusitis and otitis media with intralesional *Nasitrema* sp. and *Stenurus* sp.; mild lymphoplasmacytic meningitis; suppurative pyelonephritis. | Infectious meningitis and pyelonephritis; Parasitic sinusitis |
| 127 | Suppurative pyelonephritis with multiple renicular infarcts and severe nephrolithiasis; mild lymphocytic meningitis; mild lymphoplasmacytic cortical adrenalitis. | Infectious pyelonephritis and meningitis (septicemia); Systemic parasitosis. |
| 128 | Acute lymphoplasmacytic interstitial pneumonia; neutrophilic tracheitis; lymphoplasmacytic pyelitis; lymphoplasmacytic periportal hepatitis with scattered hepatocellular necrosis. | Infectious pneumonia, tracheitis, pyelitis and hepatitis |
| 134 | Peritonitis; hepatic abscess; suppurative bronchopneumonia with intralesional *Nasitrema* sp. and bacteria; neutrophilic myocarditis. | Infectious bronchopneumonia and myocarditis; Hepatic abscess; Systemic parasitosis. |
| 136 | Fibrinosuppurative and necrotizing bronchopneumonia with pleuritis; necrotizing adrenalitis. | Infectious bronchopneumonia; Infectious adrenalitis; Systemic parasitosis |
| 138 | Lymphocytic meningitis; neutrophilic choroiditis; severe suppurative right otitis media with intralesional *Crassicauda* sp. and *Stenurus* sp.; fibrinoulcerative pharyngitis. | Infectious meningitis and choroiditis; Parasitic pterygoid sinusitis; Systemic parasitosis |
| 139 | Lymphoplasmacytic encephalitis; multisystemic abscesses; neutrophilic cystitis. | Infectious encephalitis; Septicemia; Systemic parasitosis |
| 148 | Granulomatous transmural enteritis with intralesional *Bolbosoma* sp. and cestodes. | Intestinal parasitosis (*Bolbosoma* sp, unidentified cestodes) |
| 153 | Hemoperitoneum; severe axial muscle atrophy; suppurative subcutaneous adenitis with necrosis and intralesional *Crassicauda* sp.; granulomatous gastritis with intralesional nematodes and focally extensive lymphoplasmacytic serositis. | Systemic parasitosis. |
| 158 | Severe axial muscle atrophy; lymphoplasmacytic periportal hepatitis with intralesional *Brachycladiidae* trematodes; lymphoplasmacytic adrenalitis; chronic ulcerative dermatitis; alveolar edema and hemorrhages. | Systemic parasitosis. |
| 165 | Lymphoplasmacytic and histiocytic encephalitis; severe, chronic atlanto-occipital osteoarthritis. | Herpesviral encephalitis; Infectious osteo-arthritis. |
| 166 | Suppurative bronchopneumonia; lymphoplasmacytic choroiditis; multicentric lymphoid depletion; cervical hematoma. | Infectious bronchopneumonia and choroiditis; Trauma |
| 168 | Lymphocytic poliomyelitis and encephalitis; lymphocytic neurohypophysitis; lymphoplasmacytic adrenalitis with cortico-medullary hemorrhages and bacterial emboli; severe fibrinosuppurative and necroticoulcerative dermatitis with intralesional bacteria; suppurative bronchopneumonia with intralesional nematodes and bacteria; proliferative balanitis with keratinocyte hydropic degeneration; ulcerative glossitis, gingivitis and stomatitis. | Septicemia; herpesviral balanitis |
| 169 | Lymphoplasmacytic meningoencephalitis; necroticoulcerative pharyngitis with hemorrhage; moderate lymphoplasmacytic metritis; ulcerative and necrosuppurative dermatitis with intralesional bacteria; acute skeletal and cardiac myodegeneration. | Infectious meningoencephalitis, dermatitis, pharyngitis, and metritis; Stranding stress syndrome; Systemic parasitosis. |
| 179 | Lymphoplasmacytic cholangiohepatitis; lymphoplasmacytic cholangiohepatitis; granulomatous gastritis with intralesional *Pholleter gastrophilus*; diffuse subcutaneous edema; pulmonary edema and hemorrhage. | Systemic parasitosis |
| 183 | Lymphoplasmacytic meningoencephalitis and poliomyelitis; fibrinonecrotizing bronchopneumonia, bronchitis and bronchial sclerosis with intralesional nematodes and bacteria; suppurative pterygoid sinusitis and otitis with intralesional nematodes. | Infectious meningoencephalitis, poliomyelitis and bronchopneumonia; parasitic pterygoid sinusitis, |
| 184 | Pyogranulomatous and necrotizing encephalitis with intralesional *Nasitrema delphini* ova; granulomatous pterygoid sinusitis with *N. delphini* ova; suppurative bronchopneumonia with intralesional nematodes; multicentric lymphoid depletion. | *Nasitrema delphini* pterygoid sinusitis and encephalitis; Systemic parasitosis. |
| 196 | Lymphoplasmacytic periportal hepatitis with centrilobular hepatocellular necrosis; pulmonary edema; focal subpleural pyogranuloma with intralesional nematodes; severe pyogranulomatous mastitis with intralesional *Crassicauda* sp. | Infectious hepatitis; parasitic pneumonia; *Crassicauda* sp. mastitis |
| 197 | Pulmonary edema and hemorrhage; granulomatous and eosinophilic fasciitis and myositis with intralesional *Crassicauda* sp. (epaxial muscles); lymphoplasmacytic periportal hepatitis; suppurative mastitis with intralesional *Crassicauda* sp. | Systemic parasitosis; Infectious hepatitis |
| 201 | Pulmonary, aortic and mitral fibrinosuppurative and necrotizing endocarditis with numerous intralesional Gram-negative bacilli; lymphoplasmacytic and histiocytic meningoencephalitis; bilateral pterygoid sinusitis, otitis media and interna with intralesional *Nasitrema* sp. | *Wohlfartiimonas chitiniclastica* septicemia; Systemic parasitosis |
