# Supplementary material for: Pathologic findings and causes of death of stranded cetaceans in the Canary Islands (2006-2012)
Source: PLoS One. 2018 Oct 5;13(10):e0204444. doi: 10.1371/journal.pone.0204444 (PMC6173391; doi:10.1371/journal.pone.0204444)
Supplement: S6 Table — (DOCX) [file pone.0204444.s006.docx]

| **No** | | **Morphologic diagnosis** | **Etiologic diagnosis** | |
| --- | --- | --- | --- | --- |
| 21 | | Pulmonary edema with keratin spicules; congestion and hemorrhage in the epidural rete and central nervous system | Fetal distress | |
| 23 | | Lymphohistiocytic bronchointerstitial pneumonia with intraalveolar meconium, keratin spicules and alveolar bacteria; acute tubulonephrosis; interrenicular hemorrhage | Fetal distress (meconium aspiration) | |
| 24 | Lymphoplasmacytic periportal hepatitis; leptomeningeal hemorrhages with scattered thrombosis; Pulmonary edema | Infectious hepatitis |  |  |
| 29 | | Pulmonary edema with alveolar histiocytosis and keratin spicules; persistent ductus arteriosus | Fetal distress | |
| 30 | | Spinal meningocele; intestinal atresia; incomplete fusion and/or development of cranial bones | Developmental anomalies (congenital malformations) | |
| 38 | | Lymphocytic interstitial pneumonia with edema, intraalveolar keratin spicules and coccobacilli; cachexia; subcutaneous edema; persistent ductus arteriosus | Fetal distress | |
| 48 | | Subcutaneous edema and hemorrhage; multifocal muscular (periscapular, cervical and cranial) hemorrhage; pulmonary edema with alveolar histiocytosis, keratin spicules and atelectasia; intravascular bacteria and systemic leukocytosis | Dystocia | |
| 88 | | Multifocal subcutaneous and muscular hemorrhage; right pterygoid sinus hemorrhage; pulmonary emphysema | Dystocia | |
| 89 | | Acute lymphocytic interstitial pneumonia with intraalveolar keratin spicules, scattered fibrin and edema; subcutaneous (thoracic, cervical and abdominal) hemorrhage; persistent ductus arteriosus | Dystocia | |
| 121 | | Pulmonary edema with intraalveolar keratin spicules and hyaline membranes | Fetal distress | |
| 132 | | Pulmonary aspiration; severe cachexia; mild eosinophilic and hemorrhagic cystitis; systemic hemorrhage | Neonatal weakness | |
| 176 | | Diffuse atelectasis; pulmonary edema with intraalveolar keratin spicules | Fetal distress | |
| 213 | | Fibrinosuppurative omphaloarteritis and phlebitis; neutrophilic omentitis; pulmonary edema and hemorrhage | Septicemia | |
|  | |  |  | |

**S6 Table**. **Main morphologic and etiologic diagnoses in animals included in ‘neonatal and perinatal pathology’.**
