# Supplementary material for: Pathologic findings and causes of death of stranded cetaceans in the Canary Islands (2006-2012)
Source: PLoS One. 2018 Oct 5;13(10):e0204444. doi: 10.1371/journal.pone.0204444 (PMC6173391; doi:10.1371/journal.pone.0204444)
Supplement: S7 Table — (DOCX) [file pone.0204444.s007.docx]

**S7 Table.** **Main morphologic and etiologic diagnoses in animals included in ‘intra- and interspecific traumatic interactions’**.

| **No** | **Morphologic diagnosis** | **Etiologic diagnosis** | |
| --- | --- | --- | --- |
| **11** | Hemoabdomen; acute skeletal rhabdomyolysis; systemic hemorrhage; cardiomyopathy | Trauma | |
| **12** | Multifocal subcutaneous and muscular hemorrhage; hemoabdomen | Trauma | |
| **20** | Multifocal linear cutaneous erosions and lacerations; systemic hemorrhage | Trauma | |
| **25** | Hemothorax; focal subacute costal osteomyelitis; multifocal muscular hemorrhage | Trauma | |
| **31** | Bilateral mandibular fracture; hemoabdomen | Trauma | |
| **40** | Multifocal cutaneous linear lacerations and incisions associated with hemorrhage | Trauma | |
| **51** | Multifocal linear cutaneous lacerations and incisions with hemorrhage; multifocal thalamic and cerebellar hemorrhage; cardiomyopathy | Trauma | |
| **52** | Parietal pleural rupture and hemothorax associated with costal fracture; pulmonary atelectasia with intraalveolar keratin spicules | Trauma | |
| **58** | Multifocal rib fracture associated with pleural rupture; hemothorax | Trauma | |
| **62** | Multifocal costal fracture associated with intercostal and pulmonary hemorrhage, and diaphragmatic perforation | Trauma | |
| **64** | Necroulcerative and pyogranulomatous glossitis and stomatitis with intralesional stingray spine | Trauma | |
| **72** | Multiple costovertebral luxation and *rete mirabile* hemorrhage | Trauma | |
| **87** | Multifocal costal fracture; multifocal muscular hemorrhage | Trauma | |
| **93** | Cranial hematoma and multifocal encephalic and spinal hemorrhage | Trauma | |
| **98** | Multifocal subcutaneous (pectoral fins) and subdural (cephalic and cranial cervical vertebral canal) hemorrhage | Trauma | |
| **102** | Systemic gas embolism with hemorrhage; undigested intact squid in the upper digestive tract. | Gas embolism | |
| **105** | Maxillary and mandibular fractures; multifocal ventroabdominal muscular hemorrhage; hemopericardium; pulmonary fat embolism | Trauma | |
| **112** | Multifocal subcutaneous costal and subscapular hemorrhage | Trauma |  |
| **113** | Multifocal subcutaneous hemorrhage; pigmentary tubulonephrosis; chronic glomerulopathy | Trauma | |
| **116** | Right temporal and tympanic fracture with suppurative sinusitis and periostitis | Trauma | |
| **119** | Multifocal subcutaneous hematoma; pigmentary tubulonephrosis; Multifocal acute segmental (monophasic) myodegeneration and necrosis; Focal vena porta thrombosis | Trauma | |
| **120** | Focal melon hematoma; hydrothorax; hydropericardium | Trauma | |
| **123** | Focal thoracic subcutaneous, fascial, muscular and subpleural hemorrhage; Mild lymphoplasmacytic meningoencephalitis and myelitis; Multifocal proliferative and sclerosing bronchitis | Trauma; Infectious meningoencephalitis and myelitis | |
| **129** | Occipital fracture; hemothorax; multifocal *rete mirabile*, meningeal and aortic serosal hemorrhage | Trauma | |
| **130** | Hemopericardium; multifocal pleural and *rete mirabile* hemorrhage; Focal scapular hematoma | Trauma | |
| **135** | Multifocal vertebral and costal hemorrhage with myonecrosis; Lymphohistiocytic encephalitis; lymphohistiocytic myocarditis; systemic thrombosis with focal hepatic infarct, skeletal myonecrosis and intravascular bacteria | Polytrauma; Presumed systemic toxoplasmosis; Septicemia (*Vibrio harveyi*) | |
| **137** | Multifocal caudal vertebral fracture with periostitis, panniculitis and dermatitis; hemothorax; hemoabdomen; hemopericardium; severe pterygoid sinusitis with intralesional *Crassicauda* sp. and *Stenurus* sp.; Gas encephalopathy | Trauma;  Gas embolism | |
| **141** | Multifocal subpleural hematoma | Trauma | |
| **173** | Multifocal subcutaneous and subpleural hemorrhage; hemothorax; hemoabdomen; systemic thrombosis | Trauma | |
| **186** | Multifocal linear cutaneous lacerations with hemorrhage, soft tissue loss and osseous exposure; Multifocal cerebral and cerebellar leptomeningeal and neuroparenchyma hemorrhage; systemic fat embolism | Trauma; Fat embolism | |
| **203** | Multifocal subcutaneous and muscular hemorrhage and necrosis; bilateral multifocal costal fractures; hemothorax | Trauma | |
| **206** | Craneoencephalic trauma with intracranial, subdural and intramedullary hemorrhage; marked bilateral pulmonary emphysema | Trauma | |
| **210** | Bilateral hemothorax; costal fracture; lymphoplasmacytic meningoencephalitis, meningomyelitis and polirradiculoneuritis | Trauma; Infectious meningoencephalitis and myelitis | |
| **211** | Multifocal costal fracture associated with hemorrhage; acute skeletal rhabdomyolysis; pigmentary tubulonephrosis | Trauma | |
| **218** | Multifocal cervical subcutaneous and muscular hemorrhage; multifocal costal fracture; hemothorax; severe, chronic fibrosing arteritis with aneurysms, thrombosis and hemorrhage; severe nephritis and obstructive ureteritis with intralesional *Crassicauda* sp. | Trauma; Arterial and renal crassicaudiasis | |
| **221** | Suffusive *rete mirabile* hemorrhage; multifocal subcutaneous and muscular edema and hemorrhage; ascites | Trauma | |
| **233** | Mandibular fracture; Multifocal subcutaneous hemorrhage; Periscapular muscular tearing and myonecrosis | Trauma | |
