# Supplementary material for: Pathologic findings and causes of death of stranded cetaceans in the Canary Islands (2006-2012)
Source: PLoS One. 2018 Oct 5;13(10):e0204444. doi: 10.1371/journal.pone.0204444 (PMC6173391; doi:10.1371/journal.pone.0204444)
Supplement: S8 Table — (DOCX) [file pone.0204444.s008.docx]

**S8 Table. Main morphologic and etiologic diagnoses in animals included in ‘interaction with fishing activities’.**

| **No** | **Morphologic diagnosis** | **Etiologic diagnosis** |
| --- | --- | --- |
| **34** | Focal incisive-penetrating wounding dorsal to the right eye; Multifocal subcutaneous and intracranial hematoma and hemorrhage; lymphoplasmacytic poliomyelitis and meningoencephalitis; lymphoplasmacytic endo-myocarditis | Trauma by fishing gear; Infectious poliomyelitis and meningoencephalitis |
| **37** | Maxillary, mandibular and occipital fracture with osseous fragments penetrating the encephalic parenchyma | Trauma |
| **57** | Beak and rostral entanglement marks; Axial muscle atrophy | Entanglement |
| **66** | Penetrating cutaneous wounding with perforation of the right lung and hemothorax | Trauma by fishing gear |
| **71** | Cranioencephalic polytrauma | Trauma |
| **79** | Maxillary and occipital fracture; Mandibular fat hemorrhage; Pterygoid sinus hemorrhage | Trauma |
| **123** | Multifocal subcutaneous left thoracic hemorrhage; focal abdominal laceration with partial gastrointestinal protrusion; pulmonary fat embolism | Trauma |
| **166** | Multifocal cervical and thoracic fracture; Focal, circumferential cutaneous defect with irregular borders and underlying muscle exposure | Trauma |
| **206** | Multifocal lacerations in the rostrum, melon and ventrocaudal region with submandibular ulcers; pulmonary edema, hemorrhage, and emphysema; multifocal central nervous system meningeal and parenchymal hemorrhage and edema | Entanglement |
| **211** | Necrotizing stomatitis and mandibular osteomyelitis with intralesional hook; multifocal cutaneous incisive lesions and lacerations (anthropogenic); marked alveolar proteinosis with venous thrombosis; focal myocardial necrosis with focal coronary endarteritis and thrombosis | Trauma |
