# Supplementary material for: Pathologic findings and causes of death of stranded cetaceans in the Canary Islands (2006-2012)
Source: PLoS One. 2018 Oct 5;13(10):e0204444. doi: 10.1371/journal.pone.0204444 (PMC6173391; doi:10.1371/journal.pone.0204444)
Supplement: S9 Table — (DOCX) [file pone.0204444.s009.docx]

**S9 Table. Main morphologic and etiologic diagnoses in animals included in ‘foreign body-associated pathology’.**

| **No** | **Morphologic diagnosis** | **Etiologic diagnosis** |
| --- | --- | --- |
| **13** | Gastric perforation by linear foreign body and fibrinosuppurative peritonitis | Gastric perforation and peritonitis |
| **19** | Gastric obstruction by foreign body and hemorrhage | Gastric obstruction |
| **27** | Gastric impaction by foreign body with ulceration and hemorrhage | Gastric obstruction |
| **107** | Chronic entanglement (net lodged in the maxilla, mandible, right and dorsal pectoral fin); severe cachexia | Entanglement |
| **219** | Gastric obstruction and numerous ulcers associated with plastic bags and ropes; Pterygoid sinusitis with intralesional *Crassicauda* sp. | Gastric obstruction |
