# Supplementary material for: Pathologic findings and causes of death of stranded cetaceans in the Canary Islands (2006-2012)
Source: PLoS One. 2018 Oct 5;13(10):e0204444. doi: 10.1371/journal.pone.0204444 (PMC6173391; doi:10.1371/journal.pone.0204444)
Supplement: S10 Table — (DOCX) [file pone.0204444.s010.docx]

**S10 Table. Main morphologic and etiologic diagnoses in animals included in ‘vessel collision’.**

| **No** | **Morphologic diagnosis** | **Etiologic diagnosis** | |
| --- | --- | --- | --- |
| **3** | Left thorax cutaneous laceration with muscle shredding, tissue loss and evisceration | Trauma | |
| **10** | Cranioencephalic polytrauma; gas/fat embolism with hemorrhage | Trauma | |
| **14** | Severe scoliosis associated with deep parallel-linear cutaneous and muscle scars; severe axial muscle atrophy | Trauma | |
| **15** | Linear body section (partial amputation) with soft tissue exposure, abdominal perforation and visceral protrusion; multiple costal fractures | Trauma | |
| **32** | Linear body section (partial amputation) with abdominal perforation and visceral protrusion; fat embolism | Trauma | |
| **42** | Incisive-blunt wounding in dorsal left cranium and extensive healing wound; skeletal and myocardial rhabdomyolysis; histiocytic and neutrophilic auricular myocarditis; multifocal acute cerebral neuronal necrosis; neutrophilic adrenalitis; gas embolism | Trauma; Infectious myocarditis | |
| **47** | Complete body section (caudal amputation) | Trauma | |
| **60** | Multifocal costal and cranial fractures; hemothorax; pulmonary hemorrhage with osseous emboli | Trauma | |
| **74** | Focal cutaneous section with costal fractures, thoracic and abdominal eventration; pulmonary fat embolism | Trauma | |
| **86** | Multifocal vertebral and costovertebral fracture and hemorrhage; pulmonary fat embolism | Trauma | |
| **103** | Deep cutaneous section with muscle tearing and bone exposure at peduncle | Trauma | |
| **104** | Dorsoventral abdominal incisive-tearing wound with ‘S’ morphology, abdominal evisceration and vertebral fracture | Trauma | |
| **124** | Mandibular, condilar, supraoccipital and costal fractures; pulmonary fat embolism | Trauma | |
| **142** | Occipital incisive-tearing wounding with comminute fracture and occipital luxation; fat embolism | Trauma | |
| **150** | Thoracic and abdominal incisive wounding with abdominal eventration | Trauma | |
| **164** | Caudal fin mutilation with vertebral exposure; axial muscle atrophy | Trauma | |
| **170** | Caudal body mutilation; necrosuppurative pyelonephritis with granulomas and intralesional *Crassicauda* sp. | Trauma | |
| **182** | Thoracolumbar and diaphragmatic perforation with multiple costal fracture; chronic fibrosing endarteritis with aneurysms, thrombosis and hemorrhage; severe nephritis and obliterative ureteritis with intralesional *Crassicauda* sp. | Trauma |  |
| **190** | Peduncle amputation | Trauma | |
| **200** | Focal right retromandibular and prepectoral incisive-tearing wounding; hemothorax; left pneumothorax; hemoabdomen; focal arterial coronary thrombosis | Trauma | |
| **209** | Caudal amputation | Trauma | |
| **212** | Absence of cephalic and lumbar axial skeleton (amputation); comminute lumbar vertebral fracture; pulmonary thromboembolism with intralesional nematodes; Chronic endarteritis and granulomatous nephritis with intralesional *Crassicauda* sp. | Trauma | |
| **215** | Complete amputation of the vertebral column; Granulomatous bronchopneumonia with intralesional hyphae | Trauma;  Fungal pneumonia | |
| **216** | Left thorax incision with partial intestinal evisceration; Intracranial occipital hemorrhage | Trauma | |
